# Regulation of NKT cell-mediated immune responses to tumours and liver inflammation by mitochondrial PGAM5-Drp1 signalling

**DOI:** 10.1038/ncomms9371

**Published:** 2015-09-18

**Authors:** Young Jun Kang, Bo-Ram Bang, Kyung Ho Han, Lixin Hong, Eun-Jin Shim, Jianhui Ma, Richard A. Lerner, Motoyuki Otsuka

**Affiliations:** 1 Department of Immunology and Microbial Science, The Scripps Research Institute, La Jolla, California 92037, USA; 2 Department of Cell and Molecular Biology, The Scripps Research Institute, La Jolla, California 92037, USA; 3 Department of Gastroenterology, Graduate School of Medicine, University of Tokyo, Tokyo 113-0033, Japan

## Abstract

The receptor-interacting protein kinase 3 (RIPK3) plays crucial roles in programmed necrosis and innate inflammatory responses. However, a little is known about the involvement of RIPK3 in NKT cell-mediated immune responses. Here, we demonstrate that RIPK3 plays an essential role in NKT cell function via activation of the mitochondrial phosphatase phosphoglycerate mutase 5 (PGAM5). RIPK3-mediated activation of PGAM5 promotes the expression of cytokines by facilitating nuclear translocation of NFAT and dephosphorylation of dynamin-related protein 1 (Drp1), a GTPase is essential for mitochondrial homoeostasis. *Ripk3*^−/−^ mice show reduced NKT cell responses to metastatic tumour cells, and both deletion of RIPK3 and pharmacological inhibition of Drp1 protects mice from NKT cell-mediated induction of acute liver damage. Collectively, the results identify a crucial role for RIPK3-PGAM5-Drp1/NFAT signalling in NKT cell activation, and further suggest that RIPK3-PGAM5 signalling may mediate crosstalk between mitochondrial function and immune signalling.

## Abstract (web summary)

RIPK3-mediated signalling regulates the induction of necroptosis and inflammation. Here the authors show that RIPK3-PGAM5-Drp1 pathway is crucial for NKT cell activation independently of cell death in mouse models of melanoma and acute inflammatory liver injury.

## Graphical abstract

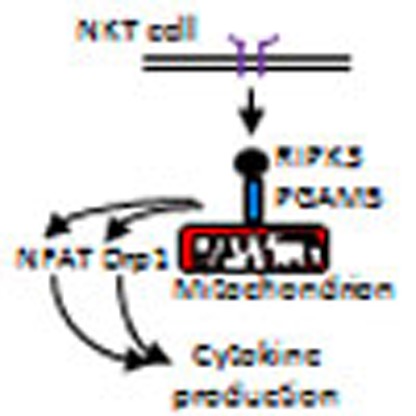

Recent studies have demonstrated that a number of genes and molecules can regulate the signalling pathways that induce necrosis. Receptor-interacting protein kinase 3 (RIPK3), is required as an upstream regulator of necroptosis, the caspase-independent form of necrosis. The release of damage-associated molecular pattern molecules (DAMPs) by necroptotic cells in turn activates innate immune cells, such as macrophages and DCs, to produce proinflammatory cytokines. Thus, RIPK3-mediated necroptosis has been suggested to be an active initiator of inflammation induced by DAMPs released from infected or damaged dying cells, and has been implicated as an underlying pathogenic mechanism for a number of inflammatory diseases^1^,^2^,^3^,^4^.

Although we do not yet have a complete understanding of the inducers and final executioners of necroptosis, the pseudokinase mixed-lineage kinase domain-like (MLKL) and the mitochondrial phosphatase phosphoglycerate mutase 5 (PGAM5) have recently been identified as key regulators of RIPK3-mediated signalling. The interaction of MLKL and RIPK3 occurs through RIPK3-dependent phosphorylation of the C-terminal kinase-like domain of MLKL^5^,^6^. MLKL has been shown to be indispensable for the induction of necroptosis, as MLKL-deficient mice are protected against necrosis-related cerulean-induced acute pancreatitis^7^. PGAM5 is recruited to the RIPK1/RIPK3 necrosome, where it is phosphorylated by RIPK3. PGAM5 further dephosphorylates and activates dynamin-related protein 1 (Drp1), a GTPase that regulates mitochondrial fission and the production of mitochondrial reactive oxygen species (mtROS). mtROS also play a role in cell death by necrosis^8^ and drive proinflammatory cytokine expression in macrophages and T-cells^9^,^10^. However, inhibition of ROS production by antioxidants does not affect cell death^11^, indicating that PGAM5 may regulate RIPK3-dependent signalling independently of ROS generation. Recent studies have provided evidence that RIPK3 independently induces inflammation without promoting cell death^12^,^13^,^14^, although the mechanism(s) by which this occurs is not clear.

Mitochondria are involved in a wide range of biological processes, such as generation of energy, biosynthesis of essential molecules and programmed cell death, and have also been implicated in the activation and control of innate and adaptive immune responses^15^. The mitochondrial protein MAVS (mitochondrial antiviral signalling protein, also known as IPS-1, Cardif, or Visa) is essential for the activation of antiviral innate immunity, and abnormal MAVS activity is often associated with the development and progression of inflammation in steatohepatitis, systemic lupus erythematosus and chronic obstructive pulmonary disease^15^,^16^,^17^,^18^. Additionally, mtROS generated by TLR signalling in macrophages activate NF-κB, MAPK, and proinflammatory cytokine production and leads to enhanced bactericidal responses^19^. In T-cells, activation of TCR signalling-dependent mitochondrial metabolism rapidly upregulates mtROS and increases production of IL-2, expansion of antigen-specific CD8^+^ T-cells, and induction of an effective memory response^20^. However, the regulatory role of mitochondria in other immune cell types has not been fully elucidated.

Type 1 natural killer T-cells (NKT cells) are CD1d-restricted, lipid antigen-reactive, and immunoregulatory T-lymphocytes. NKT cells rapidly produce pro- and anti-inflammatory cytokines upon stimulation, allowing them to regulate cellular immune responses in a broad spectrum of diseases^21^,^22^. For example, NKT cells promote the immune response to tumours and microbial infections, while suppressing immune responses in autoimmune diseases such as acute hepatitis and arthritis^21^,^22^,^23^. Furthermore, NKT cell activation can be harmful to the host in some inflammatory diseases such as atherosclerosis^24^. Despite their paradoxical functions in different disease conditions, NKT cells clearly play a crucial role in regulating the pathogenesis of various diseases.

Although RIPK3-dependent signalling in the regulation of inflammatory responses has been studied extensively in innate immune cells, little is known about its contribution to the activation of NKT cells. In this study, we demonstrate that RIPK3-mediated signalling regulates the activation of NKT cells in mouse models of melanoma and acute inflammatory liver injury. Our results indicate that RIPK3 activates PGAM5, which in turn regulates the dephosphorylation of NFAT and Drp1 to induce the expression of proinflammatory cytokines, suggesting that RIPK3-PGAM5-Drp1 signalling may play a key role in the crosstalk between mitochondrial function and signalling for NKT cell activation.

## Results

### RIPK3 regulates activation of NKT cells

**Figure 1:**
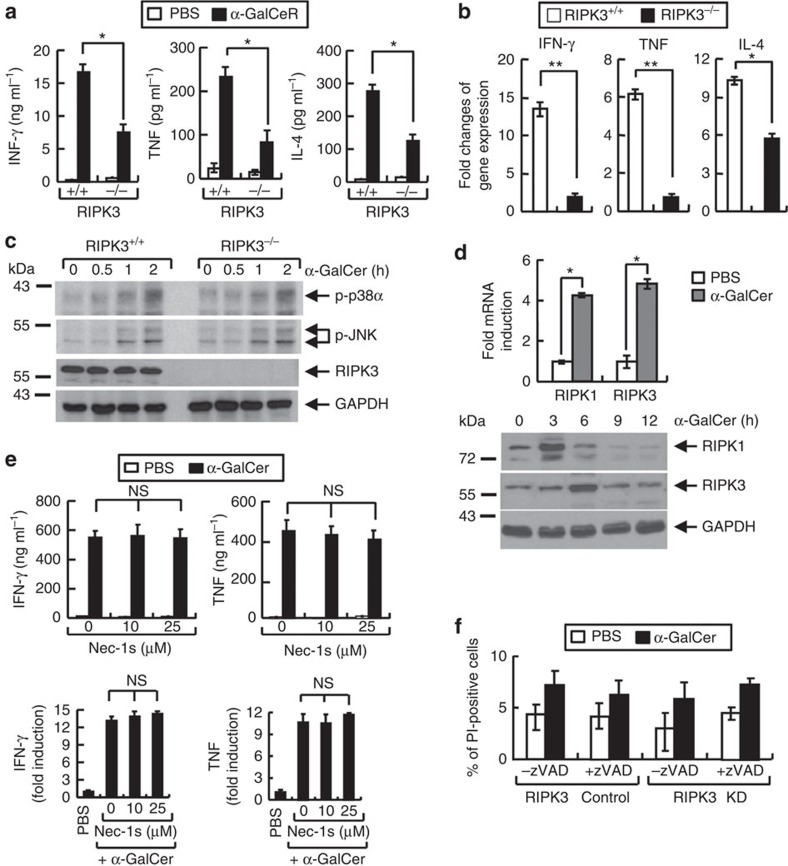
RIPK3 regulates NKT cell activation. (**a**,**b**) RIPK3-dependent expression of cytokines in NKT cells. Leukocytes isolated from the livers of WT (*Ripk3*^+/+^) or *Ripk3*^−/−^ mice were treated with PBS or α-GalCer (100 ng ml^−1^). Culture supernatants were collected at 24 h and IFN-γ and TNF concentrations were measured by ELISA (**a**), or cells were harvested at 4 h for quantification of *Ifng* and *Tnf* mRNA levels by qPCR (**b**). (**c**) Activation of MAPK signalling in NKT cells. WT or *Ripk3*^−/−^ liver leukocytes were treated with α-GalCer (100 ng ml^−1^) for the indicated times, and phosphorylation of p38α and JNK was analysed by immunoblotting. GAPDH served as a loading control. (**e**) Induction of RIPK1 and RIPK3 in NKT cells. DN32.D3 cells treated with PBS or α-GalCer (100 ng ml^−1^) for 4 h and of *Ripk1* and *Ripk3* mRNA levels were measured by qPCR analysis. DN32.D3 cells were treated with α-GalCer (100 ng ml^−1^) for indicated times, and expression of RIPK1 and RIPK3 was analysed by immunoblotting. GAPDH served as a loading control. (**e**) RIPK3-dependent NKT cell activation does not require RIPK1 activity. Liver leukocytes were incubated with PBS or α-GalCer (100 ng ml^−1^) and the indicated concentrations of the RIPK1 inhibitor Nec-1s for 24 h. Culture supernatants were collected at 24 h for quantification of IFN-γ and TNF levels by ELISA, or cells were harvested at 4 h for determination of mRNA levels by qPCR. Gene expression was normalized to levels in cells treated with PBS or medium. *Gapdh* mRNA levels were used as an internal control. (**f**) Induction of cell death. Control or *Ripk3* KD DN32.D3 cells were preincubated with PBS or zVAD (30 μM), and treated with α-GalCer (100 ng ml^−1^) for 18 h. Cells were harvested and incubated with PI, and viability was assessed by FACS analysis. Data are the means±s.e.m. (*n*=4). *P<0.005, **P<0.001 with the Mann–Whitney *U*-test; NS, not significant. Results are representative of three independent experiments. The immunoblotting experiments were repeated with new samples from 3–4 individual experiments.

Recent studies have suggested that RIPK3 can contribute to physiological and pathological immunity independently of its involvement in necroptosis^25^. A previous study has shown that RIPK3 does not regulate the activation of B cells, T-cells or macrophages^26^. Consistent with this, we have not detected an effect of *Ripk3* deficiency on cytokine production, activation of NF-κB and MAPK signalling, or proliferation in TCR-stimulated T-cells, LPS-treated B cells and TLR-stimulated peritoneal macrophages under the conditions of our experiments (Supplementary Fig. 1). Thus, our results suggest that RIPK3 signalling does not play a role in T-cells, B cells and macrophages. In this study, we asked whether RIPK3 plays a role in the activation of NKT cells *in vivo* and *in vitro*. The glycolipid α-galactosyl ceramide (α-GalCer) is a potent activator of NKT cells and induces production of regulatory and inflammatory cytokines^21^,^22^. Indeed, α-GalCer-mediated stimulation of wild-type (WT) liver leukocytes significantly increased expression of the proinflammatory cytokines IFN-γ, TNF and IL-4 at both the mRNA and protein levels (Fig. 1a,b). Unlike our observations with T-cells, B cells and macrophages, liver leukocytes isolated from *Ripk3*^−/−^ mice showed significantly reduced production of cytokines compared with WT cells (Fig. 1a,b), suggesting that RIPK3 is critical for the NKT cell cytokine expression and release by α-GalCer treatment. We next examined the activation of signalling pathways known to be essential for RIPK3-dependent cytokine production in NKT cells. Although phosphorylation and degradation of IκBα and phosphorylation of ERK were not detected, the magnitude and kinetics of p38α and JNK phosphorylation in α-GalCer-treated WT and *Ripk3*-deficient liver leukocytes were comparable (Fig. 1c). These data indicate that RIPK3 does not directly regulate MAPK-dependent signalling for inflammatory cytokine expression in NKT cells. Of note, we found that the frequency of NKT cells in spleen and liver of *Ripk3*^−/−^ mice was comparable to that of WT mice (Supplementary Fig. 2). Consistent with a previous report^27^, ∼55–60% of total lymphocyte population in isolated hepatic leukocytes was CD3^+^ lymphocyte, and 10–15% was CD3^+^CD1d/PBS57 ligand tetramer^+^ NKT cells. Although 20–30% was different types of cells such as macrophage and B cell, we could exclude their involvement because we used a NKT cell-specific ligand to induce the cell activation.

We also examined the requirement for RIPK3 in the NKT cell hybridoma line DN32.D3. Cells were infected with lentiviruses encoding control or *Ripk3*-targeting shRNA, and specific gene knockdown (KD) was verified by immunoblotting using anti-RIPK3 Ab (Supplementary Fig. 3A). As we had observed with the primary NKT cells, *Ripk3* KD significantly reduced α-GalCer-stimulated production of IFN-γ, TNF and IL-4 compared with control shRNA-expressing DN32.D3 cells (Supplementary Fig. 3B,C). Phosphorylation of p38α and JNK was comparable between α-GalCer-treated control and *Ripk3* KD DN32.D3 cells while degradation of IκBα and phosphorylation of ERK were not detected, which is similar to liver leukocytes (Supplementary Fig. 3B,C).

RIPK1 is known to regulate RIPK3 activation, and both kinases show elevated expression during cell death-associated inflammation^28^,^29^. We found that mRNA and protein levels of both kinases were significantly increased in α-GalCer-treated DN32.D3 cells (Fig. 1d); however, treatment with the RIPK1-specific inhibitor necrostatin-1s (Nec-1s)^30^ did not significantly reduce α-GalCer-stimulated expression of IFN-γ or TNF mRNA and protein (Fig. 1e). These results indicate that, despite its increased expression, RIPK1 does not play a role in RIPK3-dependent activation of cytokine production.

Next, we examined whether RIPK3 regulated necroptosis during the activation of NKT cells because RIPK3 signalling plays a key role in necroptosis in other types of cells. To determine whether the role of necroptosis, NKT cells were treated with α-GalCer plus pan-caspase inhibitor zVAD-fmk (zVAD) and viability was analysed by flow cytometry after 18 h. α-GalCer treatment did not significantly induce cell death in control and *Ripk3* KD NKT cells. The addition of zVAD did not affect the viability of control and RIPK3 KD cells, and necroptosis was not observed (Fig. 1f). These results suggest that RIPK3 regulates the activation of NKT independently of programmed cell death.

### RIPK3 promotes NKT cell-mediated anti-tumour immunity

**Figure 2:**
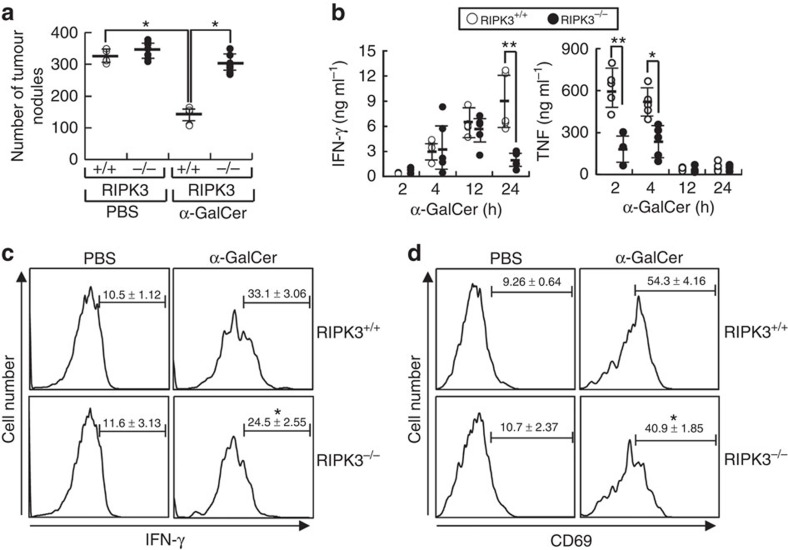
RIPK3 expressed in NKT cells is required for the anti-tumour immune response. (**a**) Enumeration of tumour nodules in the lungs of WT (*n*=6) or *Ripk3*^−/−^ mice (*n*=7). Animals were injected i.v. with B16 melanoma cells (2 × 10^5^ cells) and then injected i.p. with PBS or α-GalCer (2 μg per mouse). Lungs were collected and tumour nodules were counted 14 days later. (**b**) Production of inflammatory cytokines in WT or *Ripk3*^−/−^ mice (*n*=6). Animals were injected i.p. with PBS or α-GalCer (2 μg per mouse), and blood samples were collected at the indicated times for measurement of IFN-γ and TNF by ELISA. (**c**,**d**) Activation of NKT cells in WT or *Ripk3*^−/−^ mice (*n*=5). Animals were injected i.p. with PBS or α-GalCer (2 μg per mouse), and hepatic leukocytes were isolated 24 h later for FACS analysis of intracellular IFN-γ levels (**c**) and CD69 surface expression (**d**) in CD3+ mCD1d/PBS57 ligand tetramer+ NKT cells. Numbers are the percentage of cells within the indicated gates. Data are the means±s.e.m. (*n*=4). *P<0.005, **P<0.001 with the Mann–Whitney *U*-test; NS, not significant. Results are representative of two or three independent experiments.

NKT cells are crucial participants in the anti-tumour immune response, acting both indirectly through the production of IFN-γ and directly through induction of tumour cell lysis^31^. Administration of α-GalCer targets only NKT cells, and many investigators have used synthetic α-GalCer, or its variants to induce a strong NKT cell anti-tumour immune response in mice^32^. We used the mouse B16 melanoma model to examine the requirement for RIPK3 in NKT cell responses to tumours^22^,^23^. For this, WT or *Ripk3*^−/−^ mice were injected first i.v. with B16 melanoma cells and then i.p. with either PBS or α-GalCer, and tumour nodules in the lungs were examined. We detected a similar number of tumour nodules in the lungs of PBS-injected WT and *Ripk3*^−/−^ mice (Fig. 2a and Supplementary Fig. 4). However, administration of α-GalCer significantly reduced the number of nodules in WT mice but had no effect in *Ripk3*^−/−^ mice (Fig. 2a).

α-GalCer treatment increased the serum concentrations of IFN-γ and TNF in WT and *Ripk3*^−/−^ mice, but the levels were higher in WT compared with *Ripk3*^−/−^ mice (Fig. 2b), and intracellular expression of IFN-γ was significantly lower in liver leukocytes isolated from α-GalCer-treated *Ripk3*^−/−^ mice than from similarly treated WT mice (Fig. 2c). Also, reduction of the activation marker CD69 induction was statistically significant in liver leukocytes of α-GalCer-treated *Ripk3*^−/−^ mice (Fig. 2d). Collectively, these results strongly suggest that RIPK3 plays a crucial role in the NKT cell-mediated anti-tumour immune response.

### RIPK3 deficiency attenuates NKT cell-mediated inflammation

**Figure 3:**
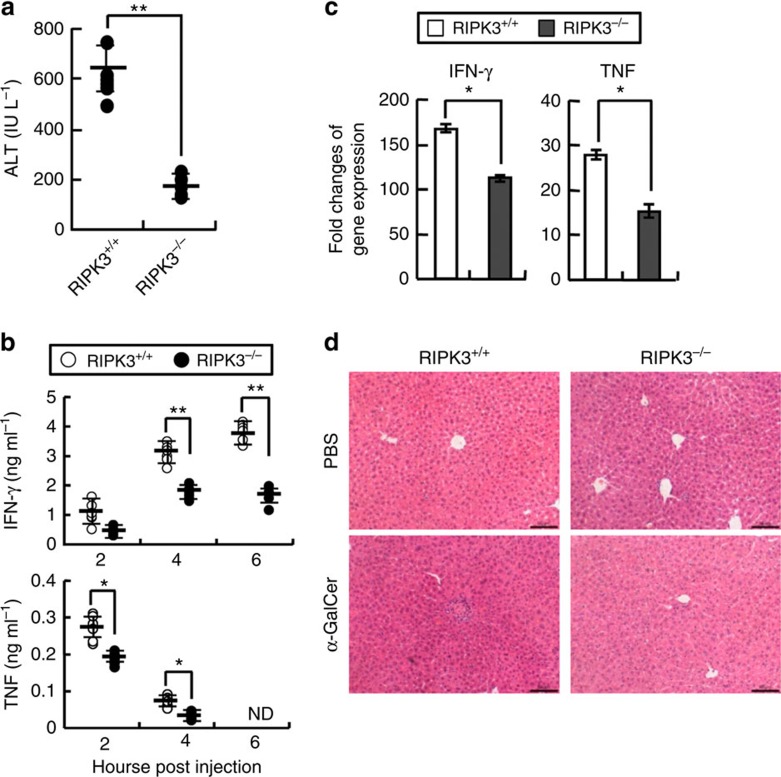
RIPK3 regulates α-GalCer-induced NKT cell-mediated inflammatory responses *in vivo*. (**a**,**b**) Groups of WT or *Ripk3*^−/−^ mice (*n*=6) were injected i.v. with PBS or α-GalCer (2 μg per mouse), and blood samples were collected 12 h later for measurement of ALT levels (**a**) or 2, 4 and 6 h later for measurement of IFN-γ and TNF levels (**b**). (**c**) qPCR analysis of *Ifng* and *Tnf* mRNA levels in the livers of WT or *Ripk3*^−/−^ mice (*n*=6) at 4 h after injection of PBS or α-GalCer (2 μg per mouse). Gene expression was normalized to levels in the livers of PBS-injected mice. (**d**) H&E staining of liver specimens from WT or *Ripk3*^−/−^ mice prepared 12 h after injection of PBS or α-GalCer. Bars=40 μm. Data are the means±s.e.m. *P<0.005, **P<0.001 with the Mann–Whitney *U*-test; ND, not detected. Results are representative of two to four independent experiments.

Given the marked effects of RIPK3 deficiency on NKT cell production of TNF and IFN-γ in the melanoma model, we next examined RIPK3 involvement in a model of inflammatory tissue damage. Previous studies have shown that NKT cells contribute to the development of acute liver damage, as evidenced by the resistance to acute Con A-induced hepatitis of *Ja18*^−/−^ and *CD1d*^−/−^ mice, which lack NKT cells^33^,^34^. Moreover, administration of α-GalCer alone induces liver injury by activating NKT cells^35^. We examined the role of RIPK3 in NKT-mediated liver inflammation by injection of α-GalCer into WT or *Ripk3*^−/−^ mice followed by examination of serum alanine aminotransferase (ALT) levels and liver pathology. Notably, although α-GalCer markedly increased serum ALT levels and liver inflammatory cell infiltrates in WT mice, liver injury was significantly lower in *Ripk3*^−/−^ mice (Fig. 3a,d), indicating that ablation of *Ripk3* protected against acute liver damage. Furthermore, α-GalCer-injected *Ripk3*^−/−^ mice showed substantially reduced levels of IFN-γ and TNF protein in the serum and mRNA in the liver compared with WT mice (Fig. 3b,c), consistent with the effects of *Ripk3* ablation on NKT cell activation. The increase in TNF levels preceded that of IFN-γ, as previously noted^33^,^36^, and this was observed whether α-GalCer was injected i.p. or i.v. (Figs 2b and 3b).

**Figure 4:**
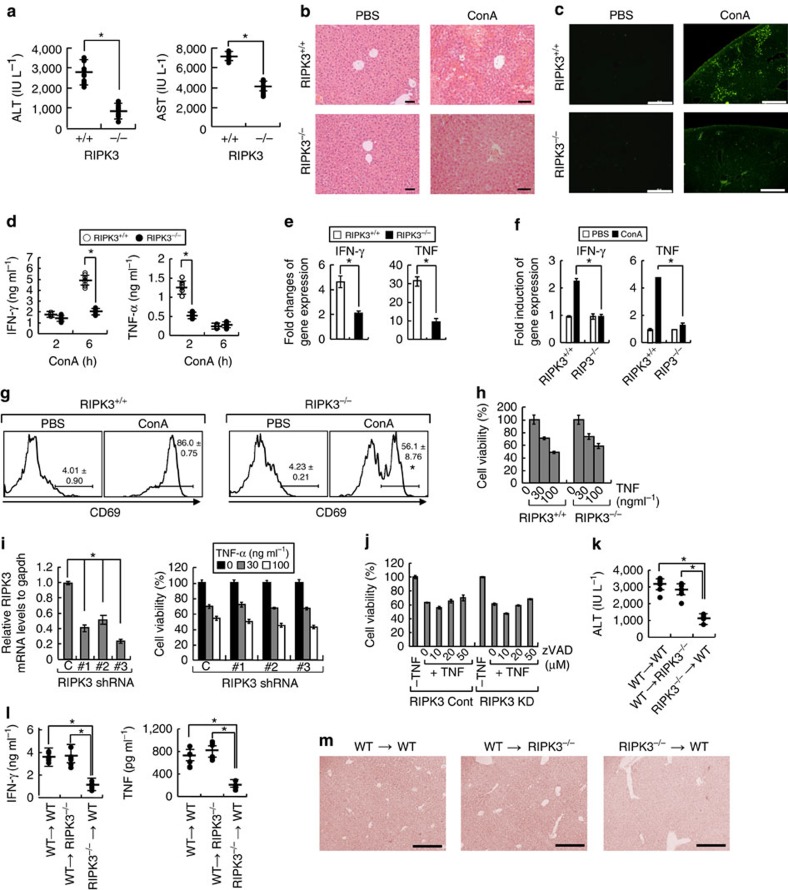
RIPK3 promotes Con A-induced liver inflammation. (**a**) Groups of WT or *Ripk3*^−/−^ mice (*n*=12) were injected i.v. with Con A, and blood samples were collected 8 h later for measurement of ALT and AST levels. (**b**) H&E staining of liver specimens from PBS- or Con A-injected mice. Bars=20 μm. (**c**) TUNEL staining of liver specimens from PBS- or Con A-injected mice. Bars=40 μm. (**d**) IFN-γ and TNF levels in blood samples collected from WT or *Ripk3*^−/−^ mice (*n*=8) at 2 or 6 h after injection of Con A. (**e**,**f**) qPCR analysis of *Ifng* and *Tnf* mRNA levels in the livers (**e**) or liver leukocytes (**f**) of WT or *Ripk3*^−/−^ mice (*n*=5) at 4 h after injection of Con A. Gene expression was normalized to the levels in livers or liver leukocytes from PBS-treated mice. (**g**) Activation of liver leukocytes from WT or *Ripk3*^−/−^ mice (*n*=5) prepared at 4 h after injection of PBS or Con A. Expression of CD69 in CD3^+^ CD1d/PBS57 ligand tetramer^+^ cells was analysed by FACS. Numbers are the percentage of cells within the indicated gates. (**h**) Hepatocytes from WT or *Ripk3*^−/−^ mice were incubated with cycloheximide (5 μg ml^−1^) and the indicated concentrations of TNF for 24 h, and cell viability was measured using the WST-1 assay. (**i**) Hepa 1–6 hepatocytes were infected with lentiviruses encoding control or three *Ripk3* shRNA (#1–3), and KD efficiency was examined by qPCR (left panel) (*n*=3). Cells were incubated with cycloheximide and TNF for 24 h before cell viability was measured (right panel) (*n*=6). (**j**) Control or *Ripk3* KD Hepa 1–6 cells were incubated with CHX, TNF-α and zVAD (30 μM) as indicated. Cell viability was evaluated after 24 h. (**k**,**l**) RIPK3 in NKT cells contributes to the acute liver injury. BM chimeric mice were generated WT→WT, WT→*Ripk3*^−/−^ and *Ripk3*^−/−^→WT (donor→recipient). After 5 weeks, mice were injected i.v. with ConA, and blood ALT levels (4 h) (**k**) or cytokine levels (2 h for TNF and 4 h for IFN-γ) were measured (**l**). (**m**) H&E staining of liver specimens from Con A-injected mice. Bars=40 μm. Data are the means±s.e.m. *P<0.001 with the Mann–Whitney *U*-test. Results are representative of at least three independent experiments.

To confirm these results, we used the ConA-induced acute liver injury model. As was observed in α-GalCer-injected mice, *Ripk3* deficiency significantly reduced the Con A-stimulated increase in serum ALT and aspartate aminotransferase (AST) concentrations (Fig. 4a). Con A-induced liver damage was also less severe in the *Ripk3*^−/−^ mice, as demonstrated by the reduction in inflammatory cell infiltrates and apoptotic cells (Fig. 4b,c). Other inflammatory markers significantly reduced in the *Ripk3*^−/−^ mice included IFN-γ and TNF protein levels in the serum and mRNA levels in the liver, and surface CD69 expression and cytokine mRNA levels in liver leukocytes (Fig. 4d–g). Collectively, these results confirm the critical role played by RIPK3 in NKT cell activation during acute liver inflammation. While *Ripk3*-deficient mice are less sensitive than WT mice to liver damage caused by α-GalCer or Con A, they are equally as sensitive to LPS/GalN-induced damage (Supplementary Fig. 5)^37^. Because innate immune cells play an important role in the LPS/GalN injection model, and RIPK3 does not regulate TLR-mediated innate immune responses^26^, this observation supports the specific involvement of RIPK3 in NKT cell-mediated immune responses. Furthermore, RIPK3 activation is independent of Fas signalling-mediated apoptosis during acute liver injury (Supplementary Fig. 5)^38^.

TNF plays an important role in the death of hepatocytes during liver injury, and consistent with this, mice lacking TNFR1 are resistant to Con A-induced liver injury and inflammation^37^. To determine whether the attenuation of α-GalCer- or Con A-induced liver damage observed in *Ripk3*^−/−^ mice is due to a lack of RIPK3 expression in hepatocytes, we directly analysed TNF-induced cell death of *Ripk3*-deficient primary hepatocytes or hepatocyte cell lines. However, we found no differences in the susceptibility of WT and *Ripk3*-deficient hepatocytes to TNF-induced death (Fig. 4h,i). These data suggest that RIPK3 expressed in NKT cells, not in hepatocytes, plays a critical role in NKT cell-mediated acute liver inflammation and injury.

Next, we tested the role of RIPK3 in the induction of necroptosis in hepatocytes. Treatment with TNF-α+CHX and zVAD did not show any significant effect on the viability of control and *Ripk3* KD hepatocytes (Fig. 4j), indicating that RIPK3-mediated necroptosis did not play a role in TNF-α-induced hepatocyte cell death. Previous studies demonstrated that RIPK3 played a critical role in the induction of programmed necrosis in many types of cells^39^,^40^. However, we did not observe any significant changes in TNF-induced cell death in *Ripk3*^−/−^ primary hepatocytes and the *Ripk3* KD hepatocyte cell line. This led us to hypothesize that the basal expression level of *Ripk3* in hepatocytes was too low to regulate the induction of cell death, which was not affected by deletion or silencing *Ripk3*. Thus, we compared the expression levels of RIPK3 in mouse tissues by qPCR analysis. Endogenous mRNA levels of *Ripk3* in heart, intestine, lung and spleen were higher than those in some tissues such as brain, liver and muscle (Supplementary Fig. 6A), which is consistent with the expression patterns of *Ripk3* in human tissues^41^. Therefore, we conclude that *Ripk3* deficiency in hepatocytes does not contribute to attenuation of acute liver damage, and TNF-induced cell death in hepatocytes is not regulated by RIPK3.

### RIPK3 in NKT cells is critical for acute liver injury

To confirm the role of RIPK3 in NKT cells during acute liver damage, we generated BM chimeric mice of the following groups (donor→recipient): WT→WT, WT→*Ripk3*^−/−^, and *Ripk3*^−/−^→WT. Mice were injected i.v. with ConA, and ALT, IFN-γ and TNF levels in the sera were measured. ConA increased serum ALT levels and infiltration of inflammatory cells in the livers of WT→WT and WT→*Ripk3*^−/−^ mice. However, liver injury was significantly lower in *Ripk3*^−/−^→WT mice (Fig. 4k,m), indicating that RIPK3 in NKT cells plays a crucial role in the induction of acute liver inflammation. Furthermore, *Ripk3*^−/−^→WT mice showed substantially reduced levels of IFN-γ and TNF in the sera compared with WT→WT and WT→*Ripk3*^−/−^ mice (Fig. 4l). Collectively, our result indicates that RIPK3 regulates the inflammatory responses of NKT cells in acute liver inflammation while RIPK3 is dispensable in hepatocytes.

### PGAM5 is the downstream regulator of RIPK3 signalling

**Figure 5:**
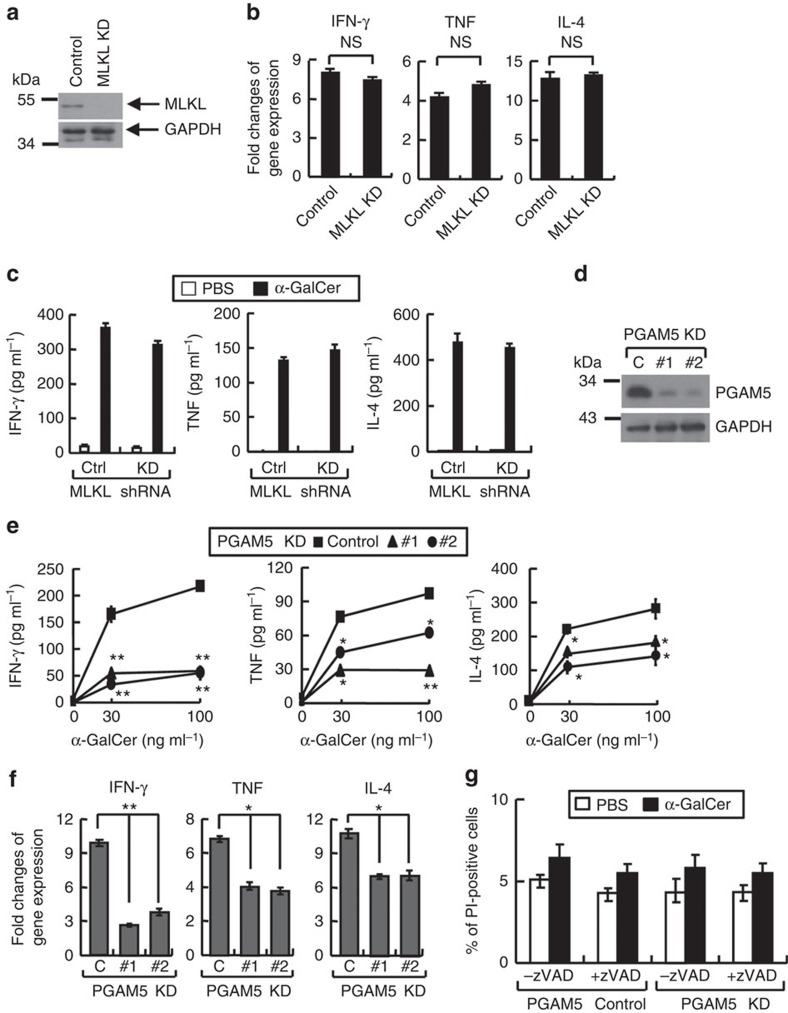
Phosphatase PGAM5 regulates NKT cell activation. (**a**–**c**) MLKL is dispensable for NKT cell activation. DN32.D3 cells were infected with lentiviruses encoding control or *Mlkl* shRNA, and KD efficiency was assessed by immunoblotting using anti-MLKL Ab. GAPDH level was measured as a loading control (**a**). qPCR analysis of *Ifng*, *Tnf* and *Il4* mRNA levels in control or *Mlkl* KD cells treated with medium or α-GalCer (100 ng ml^−1^) for 4 h (*n*=4) (**b**). (**c**) IFN-γ, TNF and IL-4 production by control or *Mlkl* KD cells treated with medium or the indicated concentration of α-GalCer for 24 h (*n*=4). (**d**–**f**) Involvement of PGAM5 in RIPK3-dependent activation of NKT cells. DN32.D3 cells were infected with lentiviruses encoding control or *Pgam5* shRNAs, and KD efficiency was assessed by immunoblotting using anti-PGAM5 Ab. GAPDH level was measured as a loading control (**d**). Control or PGAM5 KD cells treated with medium or α-GalCer (100 ng ml^−1^), and culture supernatants were collected 24 h for analysis of IFN-γ, TNF and IL-4 production (**e**) (*n*=5) or cells were harvested at 4 h for qPCR analysis of *Ifng*, *Tnf* and *Il4* mRNA (**f**) (*n*=4). (**g**) Induction of cell death. Control or *Pgam5* KD DN32.D3 cells were preincubated with zVAD (30 μM) or not, and treated with α-GalCer (100 ng ml^−1^) for 18 h. Cells were harvested and incubated with PI, and viability was assessed by FACS analysis (*n*=4). Data are the means±s.e.m. Absence of error bars indicates that they fall within the symbols. *P<0.005, and **P<0.001 with the Mann–Whitney *U*-test. N. S.=not significant. Results shown are the representative of three independent experiments. The immunoblotting experiments were repeated with new samples from 3–4 individual experiments.

MLKL and PGAM5 are both directly activated by RIPK3 and are key regulators of RIPK3-mediated signalling^5^,^6^,^8^. Additionally, RIPK3 phosphorylation of MLKL is required for RIPK3-dependent necroptosis, and PGAM5 also interacts with the RIPK3. We explore the involvement of these downstream mediators in NKT cell proinflammatory functions. Because liver leukocytes and DN32.D3 NKT cells showed the same response to α-GalCer treatment (Fig. 1 and Supplementary Fig. 3), we generated DN32.D3 NKT cells expressing *Mlkl* or *Pgam5* shRNAs to knock down the genes (Fig. 5a,d, respectively), and examined α-GalCer-stimulated expression of cytokines. IFN-γ, TNF and IL-4 mRNA and protein levels were comparable between control and *Mlkl* KD DN32.D3 cells (Fig. 5b,c) but were significantly lower in α-GalCer-stimulated *Pgam5* KD cells compared with control cells (Fig. 5e,f). These results indicate that PGAM5, but not MLKL, is an essential downstream mediator of RIPK3-dependent NKT cell activation.

We further tested whether PGAM5 regulated the induction of necroptosis in α-GalCer-treated NKT cells. Treatment with α-GalCer and zVAD did not show any significant difference in viability between control and *Pgam5* KD DN32.D3 cells (Fig. 5g), indicating that PGAM5 did not play a role in necroptosis. Collectively, these results implicate PGAM5 as a key mediator of RIPK3-mediated activation of NKT cells.

### RIPK3-PGAM5 signalling regulates the activation of NFAT

**Figure 6:**
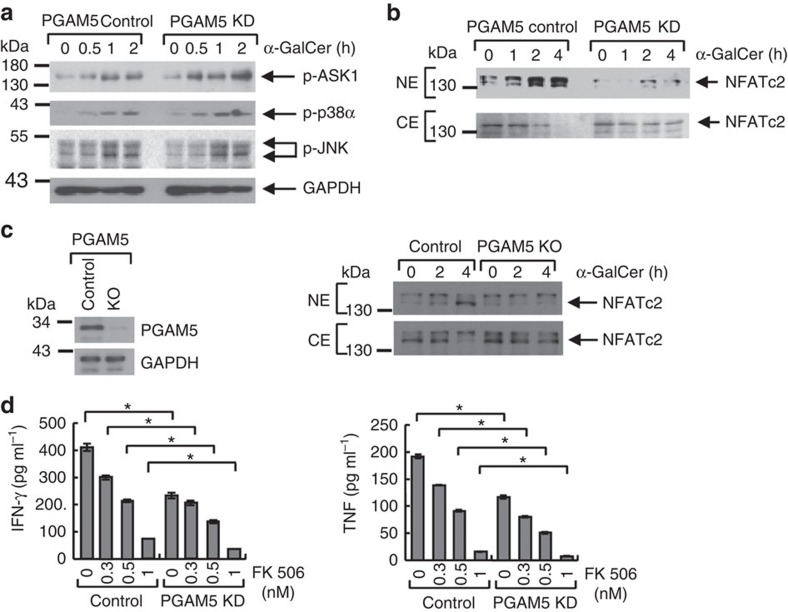
PGAM5 regulates nuclear translocation of NFAT in NKT cells. (**a**,**b**) Control or *Pgam5* KD DN32.D3 cells were treated with α-GalCer (200 ng ml^−1^) for the indicated times before cells were harvested. Whole cell lysates were immunoblotted for pASK1, p38α and pJNK (**a**), and nuclear or cytoplasmic extracts were immunoblotted for nuclear translocation of NFATc2 (**b**). GAPDH served as a loading control. (**c**) *Pgam5* KO efficiency by CRISPR/Cas9 method in DN32.D3 was assessed immunoblotting using anti-PGAM5 Ab. GAPDH level was measured as a loading control. Control or *Pgam5* KO cells were treated with α-GalCer (200 ng ml^−1^) for the indicated times, and nuclear or cytoplasmic extracts were immunoblotted for nuclear translocation of NFATc2. (**d**) PGAM5 and calcineurin independently regulate cytokine production by NKT cells. IFN-γ and TNF production by control or *Pgam5* KD DN32.D3 cells was assessed after 24 h treatment with α-GalCer and the indicated concentration of FK 506. Data are the means±s.e.m. (*n*=5). *P<0.005 with the Mann–Whitney *U*-test. Results are representative of at least three independent experiments. The immunoblotting experiments were repeated with new samples from 3–4 individual experiments.

Previous studies have shown that PGAM5 induces stress-activated MAPK signalling pathways by dephosphorylating and activating the apoptosis signal-regulating kinase 1 (ASK1), which in turn activates downstream p38α and JNK MAPKs^42^. We investigated the involvement of MAPK pathways in RIPK3-PGAM5-mediated inflammatory responses by examining ASK1, p38α and JNK phosphorylation in α-GalCer-treated control and *Pgam5* KD DN32.D3 cells (Fig. 6a). However, activation of these enzymes was comparable in both cell types, indicating that the ASK1-MAPK cascade is not involved in RIPK3-PGAM5-mediated NKT cell activation.

Expression of proinflammatory cytokines in NKT cells is induced by the transcription factor NFAT^43^, which is dephosphorylated in the cytoplasm and translocates to the nucleus to initiate gene transcription. Examination of nuclear extracts indicated that α-GalCer treatment markedly increased translocation of NFAT in control DN32.D3 cells but had only a modest effect on *Pgam5* KD cells (Fig. 6b), implicating PGAM5 in the regulation of NFAT activity. To confirm this result, CRISPR/Cas9-mediated gene targeting method was used to delete *Pgam5* in DN32.D3 cells. Consistent with *Pgam5* KD result, nuclear translocation of NFAT was significantly increased in α-GalCer-treated control NKT cells, but not in *Pgam5* KO cells (Fig. 6c). Because nuclear translocation of NFAT is also controlled by the Ca^2+^/calmodulin-dependent phosphatase calcineurin^44^, we examined the effect of the calcineurin inhibitor FK 506 on PGAM5-regulated activation of NFAT. Intriguingly, FK 506 not only reduced the production of IFN-γ and TNF by control cells, substantiating the importance of NFAT activation in cytokine production in NKT cells, but also further reduced cytokine production in *Pgam5* KD cells (Fig. 6d). These data thus suggest that PGAM5 and calcineurin independently regulate the activation of NFAT.

We also tested whether PGAM5 played a role in T-cells. Control or *Pgam5* KD Jurkat T-cells were incubated with anti-CD3/CD28 Abs, and cytokine production was examined. Unlike NKT cells, IL-2 and IL-4 levels were comparable between TCR-stimulated control and *Pgam5* KD Jurkat cells, while production of IFN-γ was significantly reduced in *Pgam5* KD Jurkat cells compared with control cells (Supplementary Fig. 7A,B). Additionally, nuclear translocation of NFAT was comparable between anti-CD3/28 Abs-treated control and *Pgam5* KD cells (Supplementary Fig. 7C). These data suggest that PGAM5 functions differentially in T cells and NKT cells.

### Drp1 regulates NKT cell activity independently of NFAT

**Figure 7:**
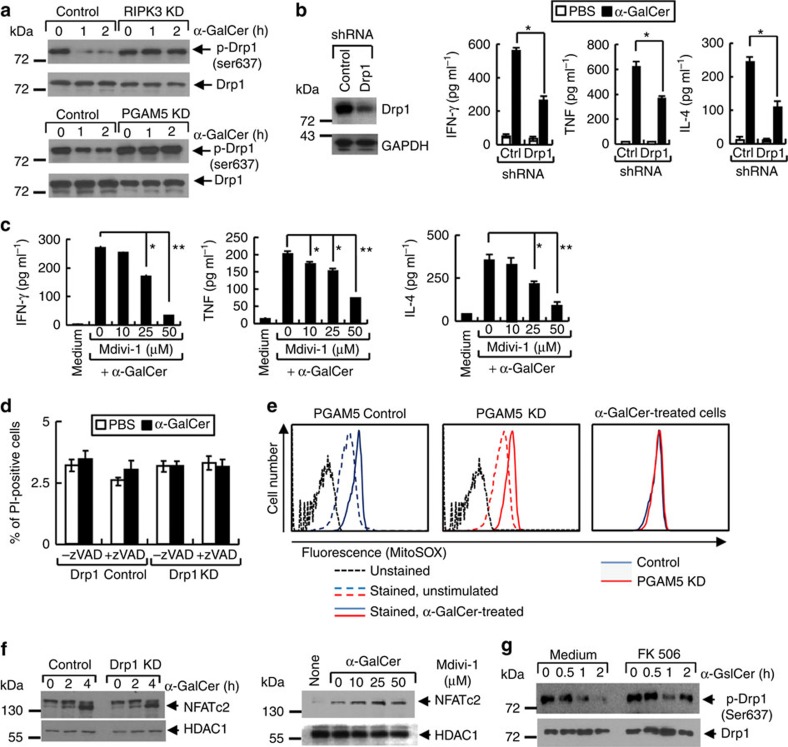
Regulation of RIPK3-PGAM5 signaling in NKT cells. (**a**) RIPK3-PGAM5 signalling regulates the dephosphorylation of Drp1. Control or *Ripk3* KD DN32.D3 cells were treated with α-GalCer, and cell lysates were immunoblotted with antibodies to total Drp1 and Drp1 (Ser637). (**b**,**c**) Drp1 is involved in cytokine production by NKT cells. (**b**) DN32.D3 cells were infected with lentiviruses encoding control or *Drp1* shRNA, and KD efficiency was examined by immunoblotting (left panel). IFN-γ, TNF and IL-4 production by control or *Drp1* KD cells was assessed 24 h after treatment with PBS or α-GalCer (right panels, *n*=6). (**c**) As described for B except cells incubated with Mdivi-1 for 30 min before addition of PBS or α-GalCer (*n*=4). (**d**) Induction of cell death. Control or *Drp1* KD DN32.D3 cells were preincubated with zVAD (30 μM) or not, and treated with PBS or α-GalCer (200 ng ml^−1^) for 18 h. Cells were harvested and incubated with PI, and viability was assessed by FACS analysis (*n*=4). (**e**) Production of mtROS in α-GalCer-treated NKT cells. Control or *Pgam5* KD DN32.D3 cells were preincubated with MitoSOX for 30 min, and treated with α-GalCer for 30 min. Cells were washed, and analysed by FACS (*n*=4). (left panel) Control cells±α-GalCer; (middle panel) *Pgam5* KD cells±α-GalCer; and (right panel) overlay of α-GalCer-treated control and *Pgam5* KD cells. (**f**) (left panel) Control or *Drp1* KD cells were incubated with α-GalCer for the indicated times, and nuclear extracts were immunoblotted with antibodies to NFATc2. HDAC1 served as a loading control for nuclear extracts. (right panel) Cells were treated as in (**c**) and nuclear extracts were immunoblotted with antibodies to NFATc2 or HDAC1. (**g**) Dephosphorylation of Drp1 by calcineurin. DN32.D3 cells were preincubated with FK 506 (1 μM) for 1 h, treated with α-GalCer for indicated times, and cell lysates were then immunoblotted with antibodies to pDrp1 (Ser637) to detected Drp1 dephosphorylation. Data are the means±s.e.m. In (**c**) absence of error bars indicates that they fall within the symbols. *P<0.005, **P<0.001 with the Mann–Whitney *U*-test. Results are representative of two or three independent experiments. The immunoblotting experiments were repeated with new samples from 3–4 individual experiments.

The PGAM5 substrate Drp1 is a GTPase that regulates mitochondrial fission and has been shown to play a role in the expression of cytokines in macrophages and T-cells^9^,^10^. Therefore, we next tested whether Drp1 contributes to RIPK3-PGAM5-mediated inflammatory cytokine expression in NKT cells. Drp1 activity and translocation to the mitochondria is regulated by dephosphorylation. We observed that dephosphorylation of Drp1 serine 637 was increased by α-GalCer treatment in control DN32.D3 cells, but not in *Ripk3* KD or *Pgam5* KD cells (Fig. 7a), demonstrating that RIPK3-PGAM5 signalling regulates the phosphorylation state of Drp1 in NKT cells.

In addition, Drp1 activity was essential for α-GalCer-stimulated cytokine production. Inhibition of Drp1 by shRNA-mediated KD or treatment with the small molecule inhibitor Mdivi-1 significantly reduced cytokine production in α-GalCer-treated NKT cells (Fig. 7b,c), suggesting that PGAM5-Drp1 signalling plays a crucial role in NKT cell activation.

Next, we compared the role of Drp1 in the activation of T-cells and NKT cells. A study has shown that Drp1 regulates cytokine production in PMA+ionomycin-treated T-cells^10^. Consistently, inhibition of Drp1 by Mdivi-1 resulted in the reduced production of cytokines and nuclear translocation of NFAT in anti-CD3/28 Abs-treated mouse T-cells (Supplementary Fig. 8). Although Drp1 regulated the activation of both T-cell and NKT cells, the upstream signalling pathways such as RIPK3 and PGAM5 that regulate the Drp1 activity are different. Therefore, these results suggest that RIPK3→PGAM5→Drp1 signalling cascade is crucial in NKT cell-mediated immune responses.

We further tested whether Drp1 affected the induction of cell death of NKT cells. Treatment with α-GalCer did not induce significant cell death in control and *Drp1* KD DN32.D3 cells, and induction of necroptosis was not affected (Fig. 7d), indicating that Drp1 does not play a role in the induction of cell death in NKT cells.

Recent studies have suggested that, in addition to the involvement of Drp1 in mitochondrial fission and fusion^45^, dephosphorylation of Drp1 by activated PGAM5 induces mtROS generation^8^,^45^. Because mtROS can act as signalling molecules for induction of cytokine gene expression^9^,^10^, we asked whether PGAM5 and/or Drp1 regulates mtROS generation and subsequent activation of NFAT in NKT cells. However, basal and α-GalCer-stimulated levels of mtROS were comparable in control and *Pgam5* KD DN32.D3 cells (Fig. 7e). Furthermore, nuclear translocation of NFAT stimulated by α-GalCer occurred normally in *Drp1* KD or Mdivi-1-treated cells (Fig. 7f), even though cytokine production was inhibited (Fig. 7b,c). From these results, we conclude that PGAM5-Drp1 signalling regulates cytokine expression independently of mtROS activity.

We also asked whether Drp1 is dephosphorylated by calcineurin in NKT cells, as has been shown in other cell types^46^. Indeed, Drp1 dephosphorylation in α-GalCer-treated cells was reduced by the addition of FK 506 (Fig. 7g). Collectively, these data suggest that in NKT cells (i) calcineurin regulates NFAT and Drp1 activities independently of RIPK3-PGAM5-mediated signalling and (ii) PGAM5 regulates NFAT and Drp1 activities independently of calcineurin activation and mtROS production.

### Inhibition of Drp1 activity protects the acute liver injury

**Figure 8:**
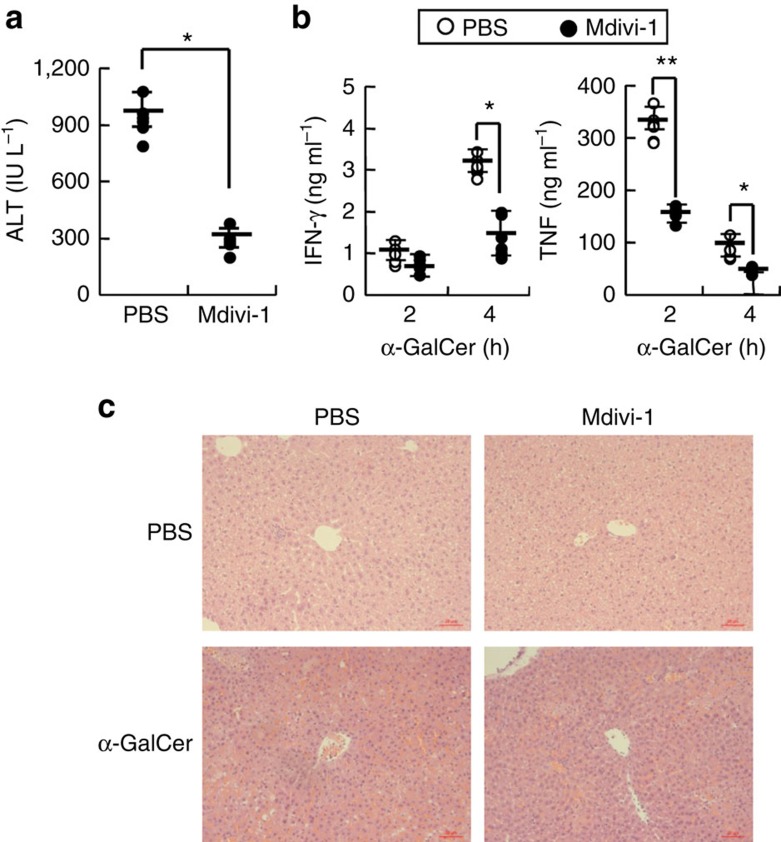
Inhibition of Drp1 activity ameliorates acute liver inflammation. (**a**,**b**) Two groups of WT mice (*n*=6 each) were injected i.p. with PBS or Mdivi-1 (25 mg per kg body weight) and then i.v. with α-GalCer (2 μg per mouse). Blood samples were collected at 12 h for measurement of ALT levels (**a**) or at 2 and 4 h for measurement of IFN-γ and TNF levels (**b**). (**c**) H&E staining of liver specimens collected 12 h after treatment as in (**a**,**b**). Bars=40 μm. Data are the means±s.e.m. *P<0.005, **P<0.001 with the Mann–Whitney *U*-test.

Our *in vitro* results have shown that Drp1 plays a regulatory role in the activation of NKT cells. To test this *in vivo*, we examined the effect of the Drp1 inhibitor Mdivi-1 on NKT cell-mediated acute liver damage induced by α-GalCer injection. Animals administered Mdivi-1 prior to α-GalCer had significantly lower serum ALT, TNF and IFN-γ levels compared with the vehicle (PBS)-injected mice (Fig. 8a,b). Additionally, mice in which Drp1 activity was inhibited showed less severe liver pathology, as indicated by reduced inflammatory cell infiltration (Fig. 8c). These results support a role for Drp1 in the activation of NKT cells during acute liver inflammation and injury.

### TCR signalling regulates RIPK3-mediated NKT cell activation

**Figure 9:**
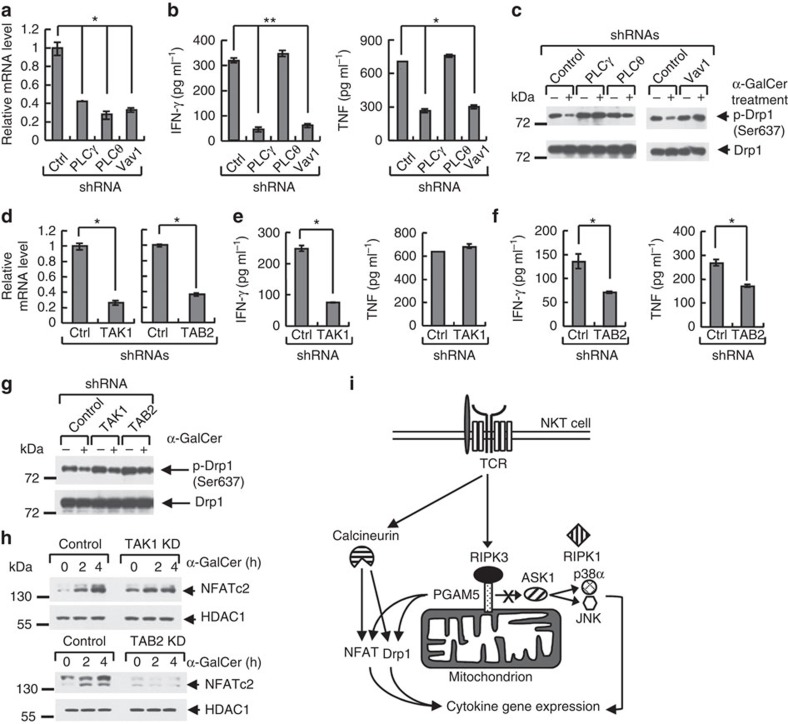
TCR-dependent activation of RIPK3-PGAM5 signalling in NKT cells. (**a**–**c**) Involvement of TCR signaling molecules in PGAM5-mediated NKT cell activation. (**a**) DN32.3 cells were infected with lentiviruses encoding control or *Plcg*-, *Pkcq*- or *Vav1*-specific shRNAs, and KD efficiency was assessed by qPCR analysis. (**b**) IFN-γ and TNF production by control and KD cells was assessed 24 h after treatment with α-GalCer (*n*=4). (**c**) Control and KD cells were treated for 1 h with α-GalCer (200 ng ml^−1^), and Drp1 dephosphorylation was analysed by immunoblotting with anti-Drp1 (Ser637) Ab. (**d**–**g**) TAB2 regulates the RIPK3-PGAM5-mediated NKT cell activation. DN32.3 cells were infected with lentiviruses encoding control or *Tak1*- or *Tab2*-specific shRNAs, and KD efficiency was assessed by qPCR. *Gapdh* levels from each cell were measured as an internal control (**d**). (**e**,**f**) IFN-γ and TNF production by control and *Tak1* KD cells (**e**) or *Tab2* KD cells (**f**) was measured after 24 h of α-GalCer treatment (*n*=4). (**g**) Drp1 dephosphorylation was assessed in control, *Tak1* KD or *Tab2* KD cells treated for 1 h with α-GalCer. (**h**) Nuclear translocation of NFAT was analysed in control, *Tak1* KD or *Tab2* KD cells at 2 or 4 h after treatment with α-GalCer. (**i**) A model of RIPK3-PGAM5-Drp1-mediated NKT cell activation. Data are the means±s.e.m. *P<0.005, **P<0.001 with the Mann–Whitney *U*-test. Results are representative of at least three independent experiments. The immunoblotting experiments were repeated with new samples from 3–4 individual experiments.

Finally, we investigated whether TCR-dependent signalling regulates RIPK3-PGAM5-Drp1-mediated NKT cell activation. For this, α-GalCer-stimulated cytokine production was analysed in DN32.D3 cells expressing shRNA specific for phospholipase C γ (*Plcg*), protein kinase C θ (*Pkcq*) or *Vav1* (Fig. 9a). Production of IFN-γ and TNF was significantly reduced by *Plcg* and *Vav1* KD, but not by *Pkcq* KD (Fig. 9b), indicating that NKT cell cytokine production occurs independently of PKCθ activity. We also examined dephosphorylation of Drp1 in these cells and found that *Plcg* or *Vav1*KD significantly reduced α-GalCer-induced Drp1 dephosphorylation, whereas *Pkcq* KD had no effect (Fig. 9c). It should be noted that we were unable to directly assay RIPK3 activity in NKT cells, because published methods for *in vitro* kinase assays of an endogenous RIPK3 are not well established but the activity of overexpressed RIPK3 in HEK 293T cells has been tested, which is not compatible with our study about the activation of NKT cell-specific signalling pathway. In addition, an anti-phospho-RIPK3 antibody is not available. Nevertheless, the results of the cytokine production and Drp1 dephosphorylation assays are consistent with important roles for PLCγ and Vav1, but not PKCθ, in RIPK3-PGAM5-Drp1-mediated inflammatory responses of NKT cells.

TCR-mediated PLCγ and Vav-1 signalling activates TGF-β-activated kinase 1 (TAK1), which plays an essential role in integrating TCR signalling for the regulation of T-cell development and activation. TAK1 acts upstream of RIPK3 to regulate its activity^47^, and activation of TAK1 requires the adapter protein TAK1-binding protein 2 (TAB2)^48^,^49^. We therefore tested the involvement of these molecules in regulating RIPK3-PGAM5-Drp1 signalling in NKT cells by analysing the cytokine response of *Tak1* KD and *Tab2* KD cells (Fig. 9d). Interestingly, we found that whereas IFN-γ levels were significantly reduced by *Tak1* or *Tab2* KD, TNF production was reduced only in *Tab2* KD cells (Fig. 9e,f). Additionally, α-GalCer-induced dephosphorylation of Drp1 was abolished by *Tab2* KD but was only modestly reduced by *Tak1* KD (Fig. 9g). Finally, we examined NFAT activation and found that α-GalCer-induced nuclear translocation of NFAT was significantly reduced by *Tab2* KD, whereas *Tak1* KD was without effect (Fig. 9h). Taken together, these data indicate that TAB2 plays an essential role in RIPK3-PGAM5-mediated activation of NKT cells.

Collectively, the results presented here reveal a critical role for TCR-dependent RIPK3-PGAM5-Drp1 signalling in regulating NKT cell-mediated immune responses (Fig. 9i). This signalling axis requires PLCγ, Vav-1 and TAB2 and induces the expression of proinflammatory cytokines by facilitating the nuclear translocation of NFAT and dephosphorylation of Drp1. Given that PGAM5 is localized at the outer membrane of the mitochondria, our results suggest that this phosphatase may play a pivotal role in coordinating crosstalk between mitochondrial function and cytokine production by NKT cells in diseases such as cancer and acute inflammatory disorders.

## Discussion

Mitochondria are essential for diverse biological processes, including energy generation, cell death and biosynthesis of essential molecules. Recent studies have shown that mitochondria are also involved in regulating innate immune cell- and T-cell-mediated immune responses. However, the regulatory roles of mitochondria in other immune cells, such as NKT cells, remain unclear. In this study, we have shown that the RIPK3-dependent mitochondrial phosphatase PGAM5 regulates NKT cell activation by dephosphorylation of NFAT and Drp1, which strongly suggests a previously unrecognized role for mitochondria in the regulation of NKT cell-mediated immunity.

RIPK1-RIPK3 signalling plays a crucial role in the induction of necroptosis. In macrophages, activation of TLR3 and TLR4 signalling in the presence of the pan-caspase inhibitor z-VAD-fmk drives RIPK1-RIPK3 complex-dependent programmed necrosis^50^. Although RIPK3 activity is dispensable for TCR-dependent T-cell activation and TLR response in macrophages^26^, the specific roles for RIPK3 signalling in innate immunity have recently been identified. In macrophages, the RIPK1-RIPK3 signalling activates formation of the NLRP3 inflammasome in response to RNA virus infection^13^. Additionally, RIPK3 in DCs plays a critical role in injury-induced inflammation and tissue repair^14^. However, we showed here that RIPK1 is not required for NKT cell activation; therefore, RIPK3 signalling for activation of NKT cell-mediated immune responses occurs independently of RIPK1.

NKT cells are involved in the regulation of a range of physiological and pathological immune responses, including anti-tumour, anti-microbial and autoimmune responses^21^,^22^. In the B16 melanoma metastasis and acute liver injury models examined here, *Ripk3* deficiency severely compromised the production of inflammatory cytokines by activated NKT cells, suggesting that RIPK3-mediated signalling likely plays an important pathophysiological role in regulating NKT cell responses to both endogenous antigens and exogenous pathogens. Our results are consistent with previous observations showing that deletion of RIPK3 reduces cell death and tissue damage in inflammatory disorders such as pancreatitis^14^,^40^,^51^. Ablation of RIPK3 protects mice against TNF-induced hepatitis as well as liver injury and inflammation resulting from ethanol-induced activation of necroptosis^4^,^40^. In addition, it has been shown that *Ripk3* mRNA is present in methionine-choline-deficient diet, human non-alcoholic steatohepatitis in liver, and hepatocarcinogenesis^16^,^52^,^53^,^54^, implicating that there may be little role in healthy liver, but RIPK3 function can be induced in diseased liver.

The results of this and other studies indicate that RIPK3 expression levels are cell-type dependent; *Ripk3* mRNA levels are high in lymphocytes, monocytes, and NK cells, but are very low in tissues such as brain, muscle and liver^41^. Although RIPK3 expression is upregulated in many cell types undergoing RIPK3-mediated cell death and inflammation^4^,^14^,^28^,^29^, we have found that RIPK3 levels in hepatocytes remain unchanged during acute liver injury while the protein levels increase in liver leukocytes. Since hepatocytes contain low basal levels of RIPK3, specific deletion or silencing of *Ripk3* in these cells did not affect induction of cell death. Additionally, pretreatment of Nec-1s significantly reduced cytokine production by α-GalCer-treated NKT cells. Since Nec-1s is a potent RIPK1 inhibitor, which has weaker indoleamine 2,3-dioxygenase inhibition compared with the prototype inhibitor Nec-1 (refs 30, 55), our result suggests that RIPK1 does not play a role in RIPK3-mediated immune response in NKT cells.

RIPK3 activates MLKL, a kinase critical for induction of necroptosis in many cell types. However, MLKL plays little or no role in the inflammatory responses of innate immune cells, as shown by the lack of effect of *Mlkl* deficiency on TNF- or LPS-induced activation of NF-κB, p38α, ERK, JNK and inflammatory cytokine production in macrophages^6^,^7^. Our results extend these observations by showing that RIPK3-dependent activation of PGAM5, but not MLKL, plays an essential role in the regulation of NKT cell activation. Although PGAM5 can activate ASK1 by dephosphorylation of inhibitory sites^42^,^56^, we found that ASK1-MAPK signalling is not involved in PGAM5-mediated NKT cell activation. However, we did find that PGAM5 regulates the transcription factor NFAT, which is crucial for cytokine production by NKT cells^21^,^43^,^44^. Interestingly, FK 506 treatment further reduced cytokine production by PGAM5 KD NKT cells, which suggests that PGAM5 and calcineurin independently regulate NFAT activation. Because PGAM5 is a phosphatase and regulates the nuclear translocation of NFAT like calcineurin, it is suggested that PGAM5 regulates the phosphorylation of NFAT. However, more work is necessary to better understand the mechanism by which PGAM5 regulates NFAT dephosphorylation.

Despite a previous study has indicated the role of PGAM5 in cell death^8^, others have suggested that RIPK3-PGAM5 signalling does not play a crucial role in the regulation of programmed necrosis^6^,^57^,^58^. Similarly, PGAM5-mediated NKT cell activation does not include the induction of cell death, supporting the notion that PGAM5 does not regulate the induction of necroptosis.

The PGAM5 substrate Drp1 regulates mitochondrial fission in many cell types and is also involved in regulating cytokine production by macrophages and T-cells^9^,^10^. Here, we showed that Drp1 inhibition or silencing reduced NKT cell activation, supporting a role for Drp1in PGAM5-mediated cytokine production. The mechanism by which this might occur is unclear. Drp1 inhibition or silencing reduces mtROS generation, raising the possibility that Drp1 activity is important for ROS generation and activation of downstream signalling pathways such as NF-κB and MAPK. However, Drp1 KD only modestly suppresses mtROS generation and NF-κB and MAPK activation in LPS-treated macrophages^9^,^10^. Moreover, we found that RIPK3-PGAM5 signalling is not necessary for ROS generation in α-GalCer-treated NKT cells, and Drp1 activity does not directly regulate NFAT activation. Therefore, it seems likely that RIPK3-PGAM5-dependent Drp1 activity may regulate NKT cell cytokine gene expression independently of mtROS generation. Because calcineurin also regulates the phosphorylation status of Drp1, this phosphatase could contribute to NKT cytokine production by acting as a dual regulator of NFAT and Drp1. However, the mechanism of Drp1 contribution to NKT cell-mediated immune responses is not limited to its role in gene expression, since inhibition of Drp1-mediated mitochondrial division ameliorates the pathology of NKT cell-induced acute liver inflammation. Therefore, more work is needed to better understand the Drp1-dependent cellular activation mechanism that regulates the gene expression. Interestingly, a recent study revealed a specific role for RIPK1-RIPK3-dependent phosphorylation of Drp1 serine 616 in RNA virus-induced activation of the NLRP3 inflammasome^13^, and another study demonstrated that dephosphorylation of Drp1 by PGAM5 is critical for mitochondrial fission and energy generation^59^. Our results indicate that dephosphorylation of serine 637, but not serine 616, is essential for cytokine production by activated NKT cells. These discrepant observations could be due to differences in stimuli, cells or pathological conditions between the studies.

TAK1 has previously been shown to regulate RIPK1-RIPK3 signalling^47^, and an association between TAK1 and TAB2 is critical for the induction of cytokine gene expression in T-cells and macrophages^48^,^49^. Nevertheless, we found that TAB2 is required for activation of TCR-induced RIPK3-PGAM5 signalling for cytokine expression by NKT cells, whereas TAK1 plays only a limited role.

In conclusion, we have demonstrated a key role for RIPK3-PGAM5 signalling in the regulation of NKT cell activation in disease states such as cancer and inflammatory disorders. Our finding that the mitochondrial phosphatase PGAM5 regulates NKT cell-mediated immune responses suggests that it may serve as a link between mitochondrial function and TCR signalling in these cells. Our results thus support a novel role for the RIPK3-PGAM5-Drp1 signalling axis in crosstalk between mitochondrial function and host immunity.

## Methods

### Mice

C57Bl/6 background WT mice were obtained from the mouse breeding facility at The Scripps Research Institute, and *Ripk3*^−/−^ (C57Bl/6 background) mice were obtained from Dr Dixit (Genentech, Inc.). Sex- and age-matched mice, usually at 8–12 weeks of age, were used for each experiment. Protocols for the use of animals were approved by the Institutional Animal Care and Use Committee.

### Reagents

The reagents and sources were as follows: Con A, LPS from *Escherichia coli* O111:B4, and N-galactosamine (GalN) (Sigma-Aldrich); TNF (Peprotech); α-galactosyl ceramide (α-GalCer, KRN7000) and Mdivi-1 (Enzo Life Sciences); Nec-1s (7-Cl-O-Nec-1, BioVision); FK 506 (LC Labs); cycloheximide (Calbiochem); phospho-ASK1(sc-109911, 1:500 dilution), NFATc2 (sc-7296, 1:1,000 dilution), RIPK1 (sc-7881, 1:1,000 dilution), RIPK3 (sc-47364, 1:1,000 dilution), and HDAC1 (sc-7872, 1:500 dilution) antibodies (Santa Cruz Biotechnology); anti-GAPDH antibody (MAB374, 1:5,000 dilution, Chemicon); phospho-p38α (#9211), phospho-JNK (#9251), phospho-ERK (#9101) and phospho-Drp1 (Ser637, #4867) antibodies (1:1,000 dilution, Cell Signaling Technology); anti-MLKL antibody (AP14272b, 1:1,000 dilution, Abgent); anti-Fas (clone Jo2, #554254, 5 μg per mouse, BD Pharmingen); CD3-FITC (#11–0032, 0.25 μg per 10^5^ cells), CD4-PerCP/Cy5.5 (#35–0042, 0.125 μg per 10^5^ cells), and CD69-PE antibodies (#1–0691, 0.25 μg per 10^5^ cells) (eBioscience); and MitoSOX RED (Molecular Probes). APC-mCD1d/PBS57 ligand tetramers were generously provided by the National Institute of Allergy and Infectious Disease MHC Tetramer Core Facility (Atlanta, GA, USA).

### Cell culture

HEK 293T, Hepa 1–6 (mouse hepatocyte cell line), Jurkat (human T-cell line) and B16 mouse melanoma cells were cultured in DMEM supplemented with 10% FBS. Mouse NKT hybridoma cells DN32.D3 were obtained from Dr Bendelac (University of Chicago) and were maintained in RPMI with 10% FBS. Cell viability was measured by the WST-1 cell proliferation assay (Roche) according to the manufacturer's instructions.

### Melanoma metastasis model

Mice were randomly injected i.v. with B16 melanoma cells in PBS (5 × 10^5^ cells per mouse), and PBS or α-GalCer (2 μg per mouse) was injected i.p. 1 h later. After 14 days, mice were killed and lungs were removed and fixed in Bouin's solution. The number of tumour nodules was counted under a dissection microscope in a blinded manner.

### Acute liver injury models

Mice were randomly injected i.v. via the lateral tail vein with Con A (20 mg per kg body weight) or α-GalCer (50 μg per kg). Anti-Fas antibodies diluted in PBS (1 μg per g body weight) were intraperitoneally injected. LPS (30 μg per kg) plus GalN (1,000 mg per kg) was intraperitoneally administered into mice. At the indicated times, animals were killed, blood was collected and the livers were surgically removed in a blinded manner. Serum samples were prepared and analysed for transaminase and cytokine levels.

### Generation of bone marrow chimeras

Chimeric mice were generated by total-body gamma irradiation followed by transfer of bone marrow cells. Briefly, WT and *Ripk3*^−/−^ recipient mice were randomly grouped and irradiated with a dose of 9 Gy and then i.v. injected with 1 × 10^6^ cells per mouse from WT and *Ripk3*^−/−^ mice. Mice were administered with antibiotics *ad libitum* for 2 weeks. After 5 weeks, mice were injected i.v. with ConA.

### ALT and AST assays

Serum ALT and AST levels were quantified with enzymatic assays (Pointe Scientific) according to the manufacturer’s instructions.

### Histology

Livers were removed, fixed in 4% paraformaldehyde, and embedded in paraffin. Tissue sections were stained with H&E or subjected to terminal deoxynucleotidyl transferase dUTP nick end labelling (TUNEL) staining according to the kit manufacturer’s instructions (*In Situ* Cell Death Detection Kit; Roche).

### Reverse transcription and quantitative PCR

Total RNA from liver or cells was prepared with TRIzol reagent (Invitrogen), and cDNA templates were generated using Superscript III reverse transcriptase (Invitrogen) with an oligo-dT primer, according to the manufacturer’s protocol. Real-time PCR analysis was performed using SYBR Green PCR Master Mix (Applied Biosystems). Expression of the genes of interest was normalized to the levels of *Gapdh* or *Actin* mRNA, and relative expression levels were calculated according to the *ΔΔC*_T_ method. Primer sequences used for quantitative PCR are listed in Supplementary Table 1.

### Hepatocyte preparation

Livers were perfused and digested by passing warm perfusion buffer (HBSS supplemented with 10 mM HEPES and 0.5 mM EGTA) and digestion buffer (HBSS supplemented with 10 mM HEPES and 4.12 μg ml^−1^ liberase (Roche)s) sequentially via the portal vein. Liver tissue was further gently digested and cells were passed sequentially through 100-, 70- and 40-μm cell strainers. Hepatocytes were isolated by centrifugation at 40*g* for 1 min at RT, and the cell pellet was washed with PBS three times. Purified hepatocytes were resuspended in William E medium (Quality Biological, Inc.) supplemented with 10% FBS and antibiotics, seeded on collagen-coated culture plates (50 μg ml^−1^, Stem Cell Technologies) for 1 h at RT, and then washed with PBS twice before use in experiments.

### Preparation of liver leukocytes

Blood was removed from the liver by perfusion with 10 ml of HBSS injected through the portal vein. Liver homogenates were incubated with 100 U ml^−1^ collagenase (Sigma) for 40 min at 37 °C, and the digest was passed through a 100-μm cell strainer. Cells were centrifuged at 700 *g* for 10 min at RT and the pellets were resuspended in 40% isotonic Percoll containing 100 U ml^−1^ of heparin. The suspension was centrifuged at 700 *g* for 20 min at RT and RBCs were removed from the non-parenchymal cell pellet with RBC lysis buffer.

### Flow cytometry

Liver leukocytes were stained with CD3-FITC, CD4-PerCP/Cy5.5, CD69-PE and APC-CD1d ligand tetramers, and subsequently fixed in 2% paraformaldehyde for analysis. To assess intracellular mtROS levels, cells were incubated with MitoSOX (5 μM) and α-GalCer (0.5 μg ml^−1^) for 30 min and washed before analysis. Data were acquired with a FACSCalibur (BD Biosciences) and analysed with FlowJo (TreeStar, Inc.).

### Plasmids and shRNAs

Lentiviral expression vectors for mouse RIPK3 shRNAs were obtained from Jiahuai Han^60^. The shRNA constructs were designed based on the single oligonucleotide RNA interference technology, and lentiviral vectors expressing these shRNAs were generated according to the manufacturer’s instructions (Biosettia). Oligonucleotides sequences used to generate the shRNAs are listed in Supplementary Table 2. *Pgam5* sgRNA CRISPR/Cas9 All-in-One Lentivector (Mouse target 1) was purchased from Applied Biological Materials (Cat # K4565206).

### Lentiviruses

Recombinant lentiviruses were packaged in HEK 293T cells by cotransfection of shRNA-encoding plasmids and helper plasmids such as pRSV-REV, pMDLg and pVSV-G.

### Western blotting analysis

Primary liver leukocytes and DN32.D3 cells were washed and incubated at 4 °C for 30 min in lysis buffer (50 mM Tris-Cl, pH 7.4, 150 mM NaCl, 2 mM EDTA, 1% NP-40, 0.5% sodium deoxycholate, and 0.1% SDS) containing protease inhibitor cocktail (Roche), 1 mM DTT, and 1 mM PMSF. Liver tissue samples were washed with ice-cold PBS, resuspended in lysis buffer at 4 °C, and homogenized. Cell and liver lysates were incubated for 30 min at 4 °C and centrifuged at 12,000 r.p.m. for 10 min at 4 °C. For preparation of nuclear extracts, cells were incubated in 160 μl of buffer (10 mM HEPES, pH 7.9, 10 mM KCl, 0.1 mM EDTA, 0.1 mM EGTA, 1 mM DTT and 0.5 mM PMSF) at 4 °C for 15 min. NP-40 was then added to a final concentration of 2.5%, and the tube was vigorously vortexed for 10 s. The homogenate was centrifuged at 12,000 *g* for 5 min at 4 °C, the nuclear pellet was resuspended in 40 μl of buffer (20 mM HEPES, pH 7.9, 400 mM NaCl, 1 mM EDTA, 1 mM DTT and 1 mM PMSF), and the tube was vigorously vortexed at 4 °C for 20 min. The extract was centrifuged at 12,000*g* for 5 min at 4 °C. Protein concentrations in the supernatants were determined by the Bradford method. Lysates were resolved by SDS–polyacrylamide gel, and proteins were transferred to membranes, immunoblotted with the appropriate primary (dilution 1:500–1,000) and secondary antibodies (dilution 1:5,000–10,000), and visualized by chemiluminescence. Images have been cropped for presentation. Full size images are presented in Supplementary Fig. 9.

### Measurement of cytokine concentrations

IFN-γ, TNF and IL-4 levels in sera or culture supernatants were quantified by ELISA (eBioscience).

### Statistical analysis

Data were analysed with the Mann–Whitney *U*-test. *P*<0.05 was considered significant. The statistical significance of the results was also confirmed by analysis of variance test, and the data passed the normality test. Statistical analysis was performed using Prism software (GraphPad, San Diego).

## Additional information

**How to cite this article:** Kang, Y. J. *et al*. Regulation of NKT cell-mediated immune responses to tumours and liver inflammation by mitochondrial PGAM5-Drp1 signalling. *Nat. Commun.* 6:8371 doi: 10.1038/ncomms9371 (2015).

## Supplementary Material

Supplementary Information Supplementary Figures 1-9 and Supplementary Tables 1 and 2
