# Supplementary material for: Regulation of NKT cell-mediated immune responses to tumours and liver inflammation by mitochondrial PGAM5-Drp1 signalling
Source: Nat Commun. 2015 Sep 18;6:8371. doi: 10.1038/ncomms9371 (PMC4576739; doi:10.1038/ncomms9371)
Supplement: Supplementary Information — Supplementary Figures 1-9 and Supplementary Tables 1 and 2 [file ncomms9371-s1.pdf]

Supplementary Figure 1

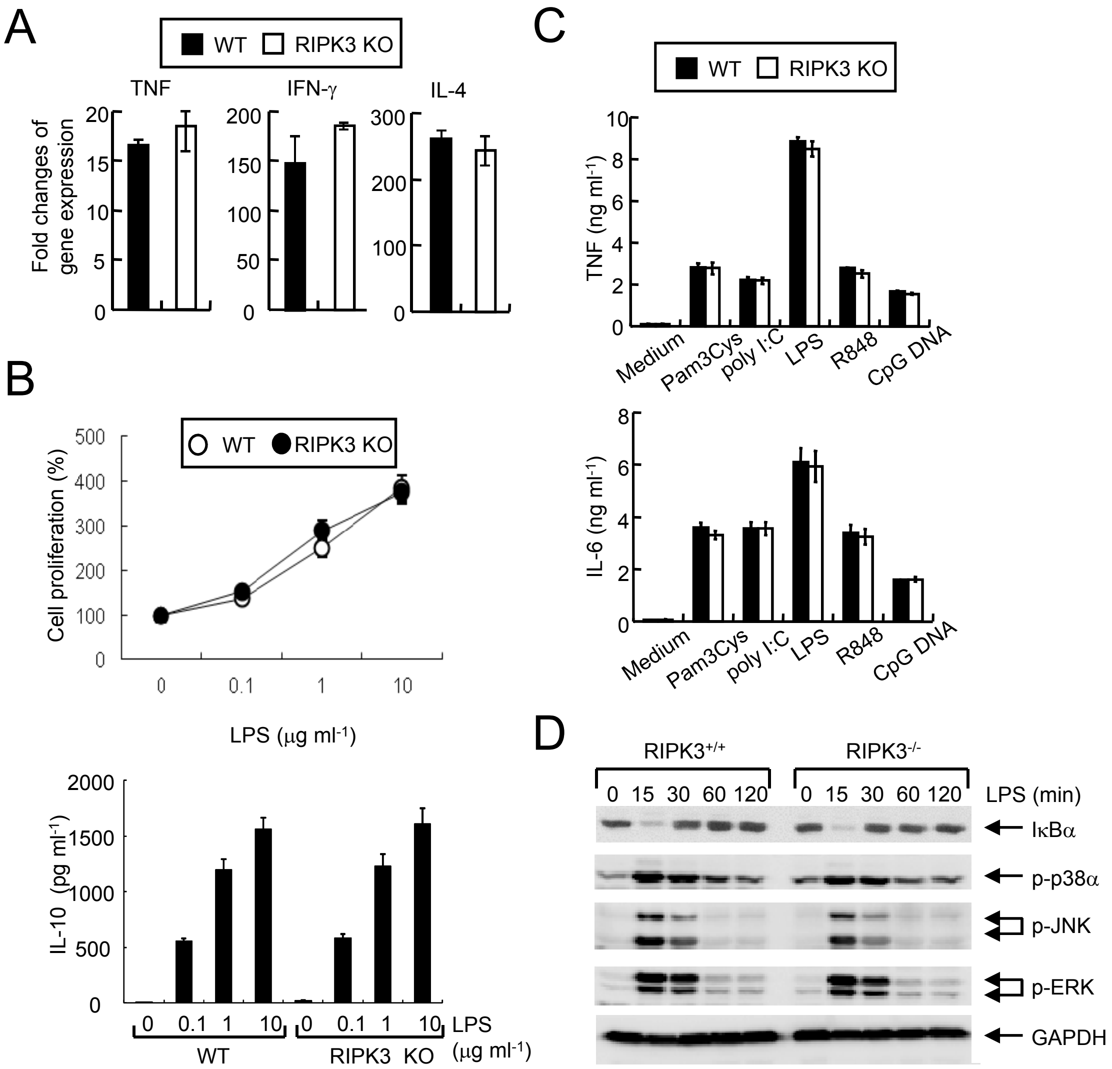

Supplementary figure 1. RIPK3 is dispensable in T cells, B cells, and macrophages.
(A) T cells were prepared from spleens of WT or *Ripk3* KO mice, and incubated in a culture plated coated with anti-CD3 (5 μg ml^{-1}) and anti-CD28 (10 μg ml^{-1}) Abs. After 8 hours, RNAs were prepared and expression cytokines was analyzed by quantitative PCR. Fold induction of gene was calculated to the expression level in unstimulated cells. (B) Purified B cells from spleens of WT or *Ripk3* KO mice were incubated with LPS, and cell proliferation was analyzed by WST-1 assay at day 2. Percentage of proliferation = (OD of LPS-treated cells/ OD of LPS-untreated cells) x 100. Production of IL-10 in culture supernatants was analyzed by ELISA after 24 h of LPS treatment. (C) Production of inflammatory cytokines by peritoneal macrophages stimulated with TLR ligands. Thioglychollate-elicited peritoneal macrophages from WT or *Ripk3* KO mice were stimulated with medium, Pam3Cys (1 μg ml^{-1}), poly I:C (50 μg ml^{-1}), LPS (0.1 μg ml^{-1}), R848 (1 μg ml^{-1}), or CpG DNA (2.5 μg ml^{-1}). Culture supernatants were obtained after 24 hours, and TNF and IL-6 levels were determined by ELISA. (D) Peritoneal macrophages from WT or *Ripk3* KO mice were stimulated with LPS, and activation of NF-κB and MAPKs was analyzed by immunoblotting. GAPDH level was probed as a loading control. Data are as mean ± s.d.

Supplementary Figure 2

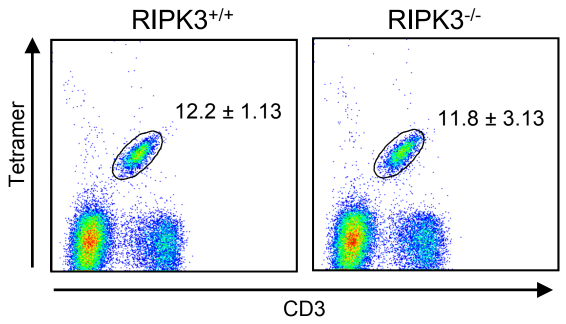

Supplementary figure 2. The number of NKT cells. Leukocytes were obtained from livers of WT and *Ripk3*$^{-/-}$ mice (n = 4). Cells were stained with anti-CD3 antibody and CD1d/PBS57 ligand tetramer. The percentage of cells in the gated area was analyzed.

Supplementary Figure 3

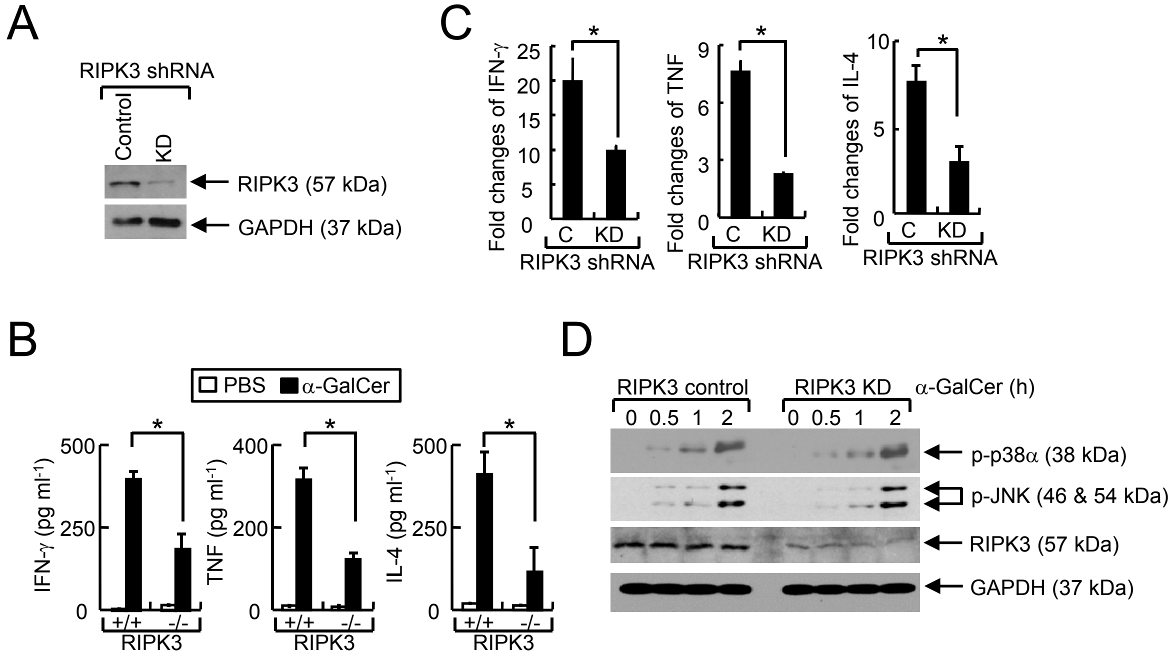

Supplementary Figure 3. RIPK3 regulates the activation of DN32.D3 NKT hybridoma cells
DN32.D3 NKT hybridoma cells were infected with control or *Ripk3* shRNA-encoding lentiviruses and efficiency of *Ripk3* KD was examined by immunoblotting (A). Control or *Ripk3* KD DN32.D3 cells were treated with a-GalCer (200 ng ml^{-1}) for the indicated times before cytokine production was measured by ELISA (B) or for 4 h to quantify mRNA levels by qPCR (C). (D) Activation of MAPK signaling in NKT cells. Control or *Ripk3* KD DN32.D3 cells were treated with α-GalCer (200 ng ml^{-1}) for the indicated times, and phosphorylation of p38α and JNK was analyzed by immunoblotting. GAPDH served as a loading control. Data are the means ± s.d. (n = 4). * $P < 0.005$, ** $P < 0.001$.

Supplementary Figure 4

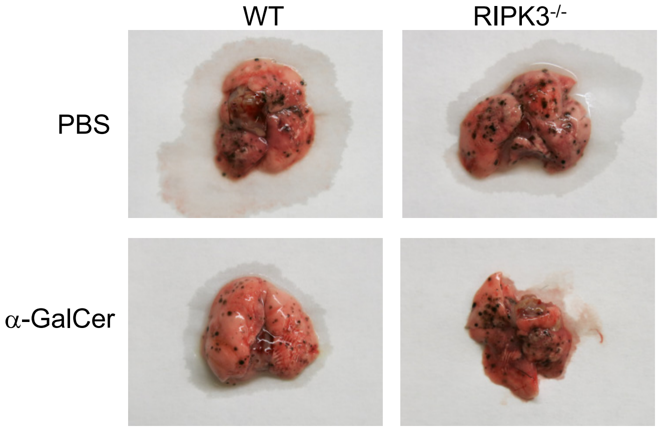

Supplementary Figure 4. Representative images of lung metastasis. WT or *Ripk3*$^{-/-}$ mice were injected i.v. with B16 melanoma cells and then injected i.p. with PBS or α-GalCer (2 μg per mouse). Lungs were collected and tumor foci were counted 14 days later.

Supplementary Figure 5

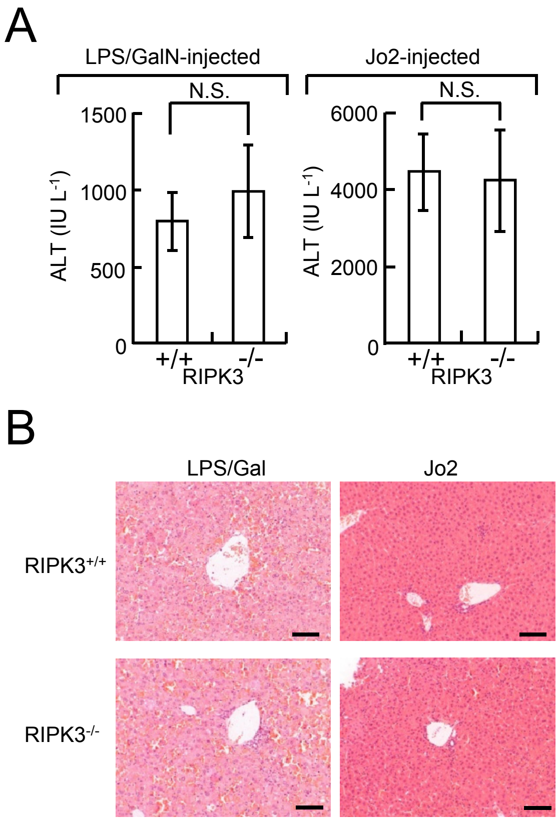

Supplementary Figure 5. RIPK3 in not involved in the liver damage induced by innate immune cells or Fas-mediated signaling. (A) WT or *Ripk3*$^{-/-}$ mice were intraperitoneally injected with LPS/GalN (n = 6) or Jo2 antibody (n = 6). Serum samples were collected after 10 h of LPS/GalN or 6 h of Jo2 antibody injection. ALT levels were measured. (B) H&E staining of liver specimens from LPS/GalN, or Jo2 antibody-injected mice. Bars, 20 μm. Data represent means ± s.d. N.S.; not significant. Results shown are the representatives of 2-3 independent experiments.

Supplementary Figure 6

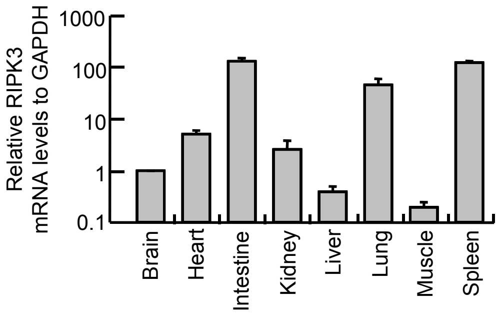

Supplementary Figure 6. Expression of RIPK3 in various tissues.
Total RNAs from mouse tissues (n=5) were prepared and expression level of *Ripk3* was analyzed by quantitative PCR. Fold induction of gene was calculated to the expression level in brain. Data are as mean ± s.d.

Supplementary Figure 7

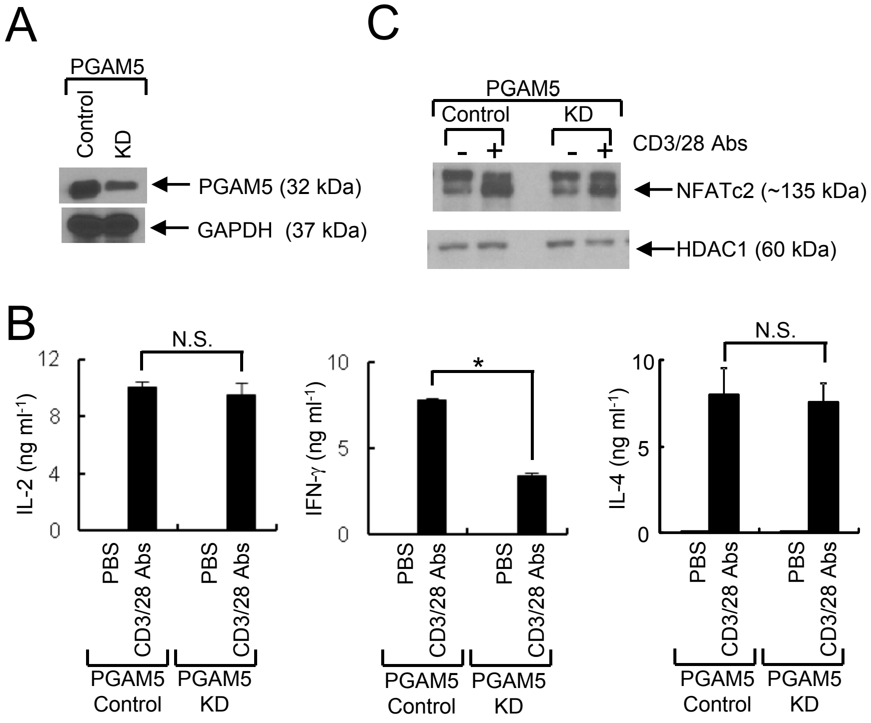

Supplementary Figure 7. PGAM5 does not regulate the activation of T cells.
Human Jurkat T cells were infected with control or *Pgam5* shRNA-encoding lentiviruses and efficiency of *Pgam5* KD was examined by immunoblotting. GAPDH served as a loading control. (A). Control or *Pgam5* KD Jurkat cells were incubated with anti-CD3 (5 μg ml^{-1}) and anti-CD28 (10 μg ml^{-1}) for 48 h, and cytokine production was measured by ELISA (B). (C) Nuclear translocation of NFAT in *Pgam5* KD Jurkat cells. Control or *Pgam5* KD Jurkat cells were incubated with anti-CD3/28 Abs for 4 h. Nuclear extracts were prepared, and translocation of NFAT was analyzed by immunoblotting. HDAC1 served as a loading control of nuclear extracts. Data are the means ± s.d. (n = 4). *P < 0.005, N.S.; not significant.

Supplementary Figure 8

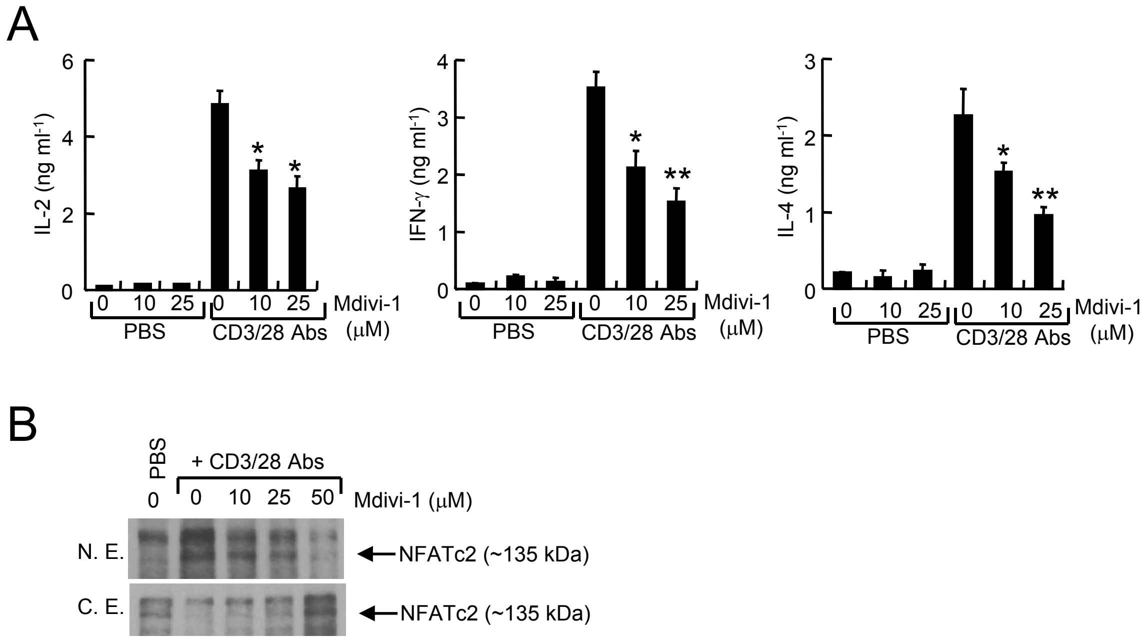

Supplementary Figure 8. Drp1 regulates the activation of T cells.
Mouse T cells were prepared from WT mouse spleen, and incubated with or without anti-CD3 (5 μg ml^{-1}) and anti-CD28 (10 μg ml^{-1}), and Mdivi-1. Culture supernatants were prepared after 24 h to analyze the cytokine levels (A), or nuclear (N.E.) and cytoplasmic extracts (C.E.) were prepared after 4 h to analyze the translocation of NFAT by immunoblotting (B). Data are the means ± s.d. (n = 4). * $P < 0.005$, ** $P < 0.001$.

Supplementary Figure 9. Uncropped images of Western blots

Fig. 1C

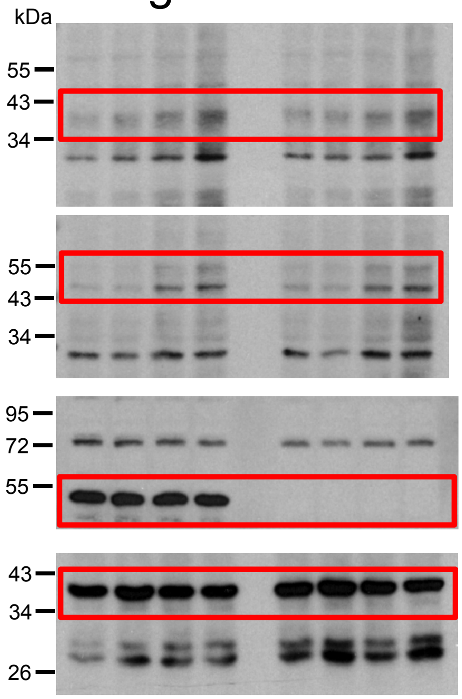

Fig. 1D

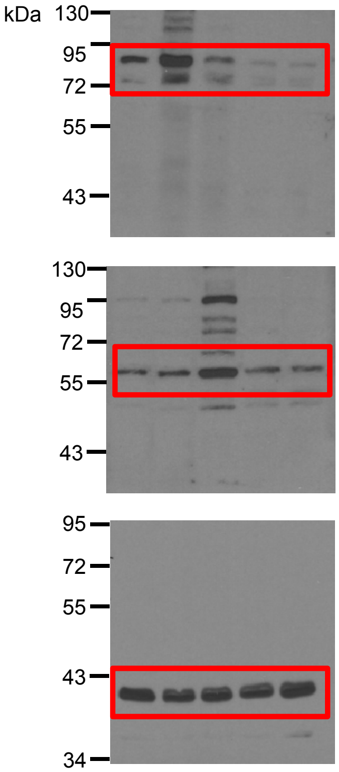

Fig. 5A

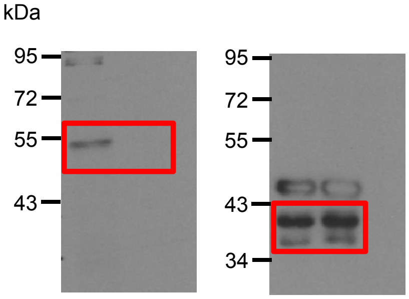

Fig. 5D

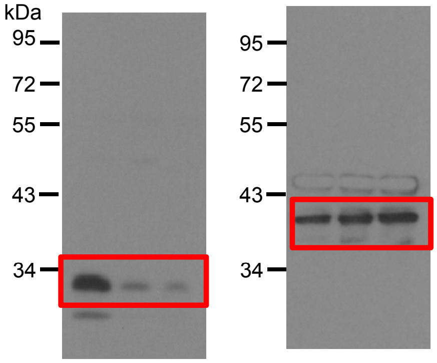

Supplementary Figure 9. Uncropped images of Western blots

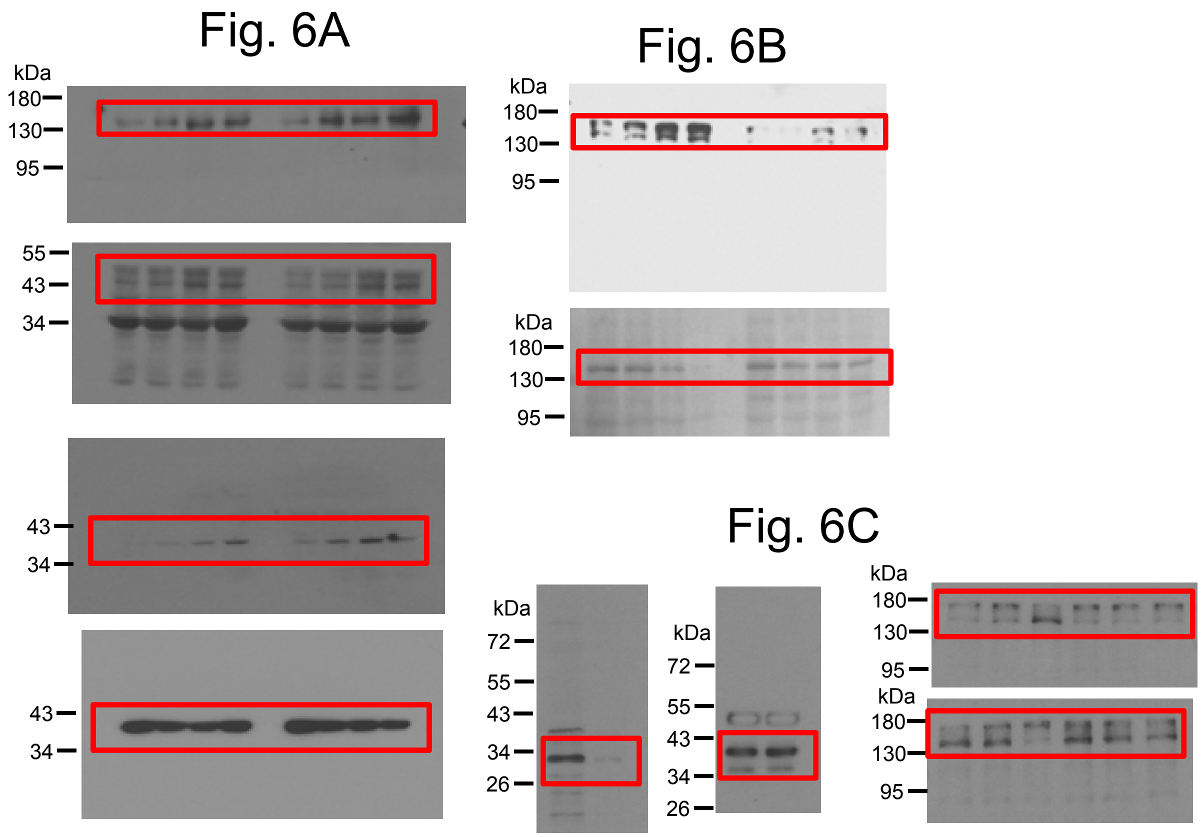

Supplementary Figure 9. Uncropped images of Western blots

Fig. 7A

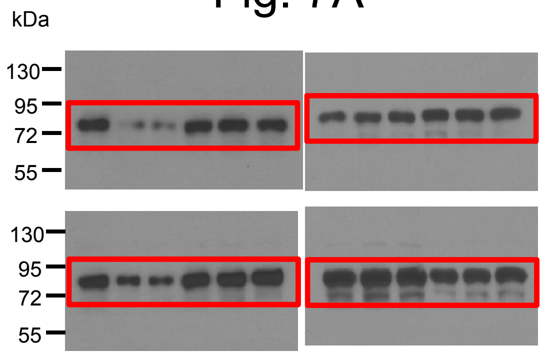

Fig. 7B

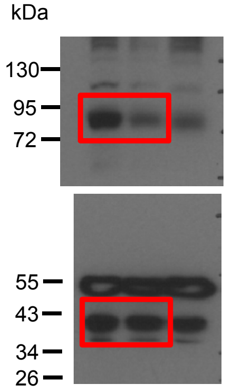

Fig. 7F

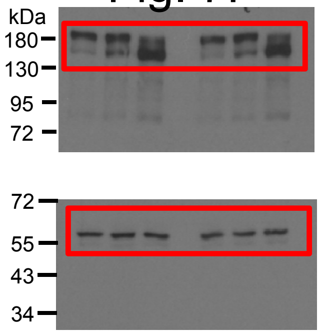

Fig. 7F

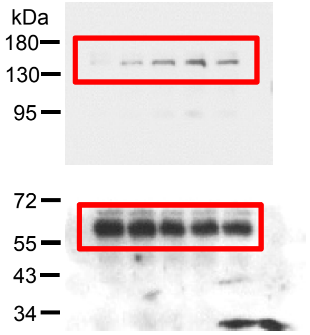

Fig. 7G

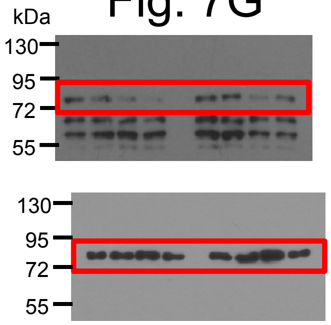

Supplementary Figure 9. Uncropped images of Western blots

Fig. 9C

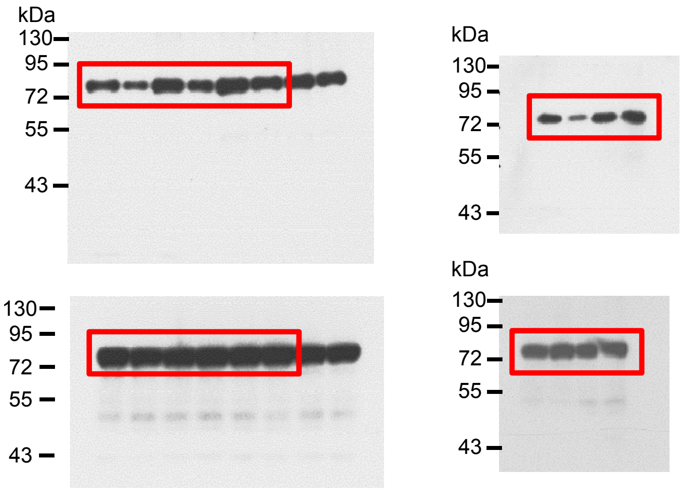

Fig. 9G

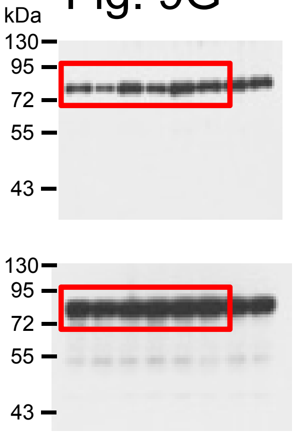

Fig. 9H

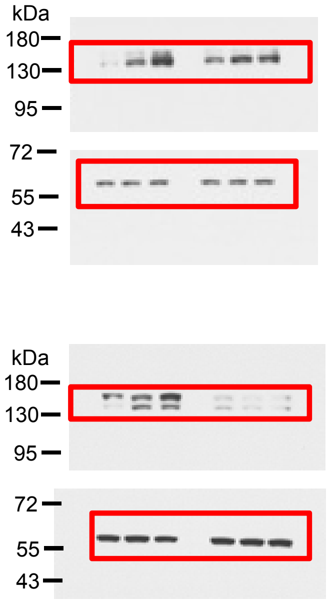

Supplementary Figure 9. Uncropped images of Western blots

Supplemental Fig. 1C

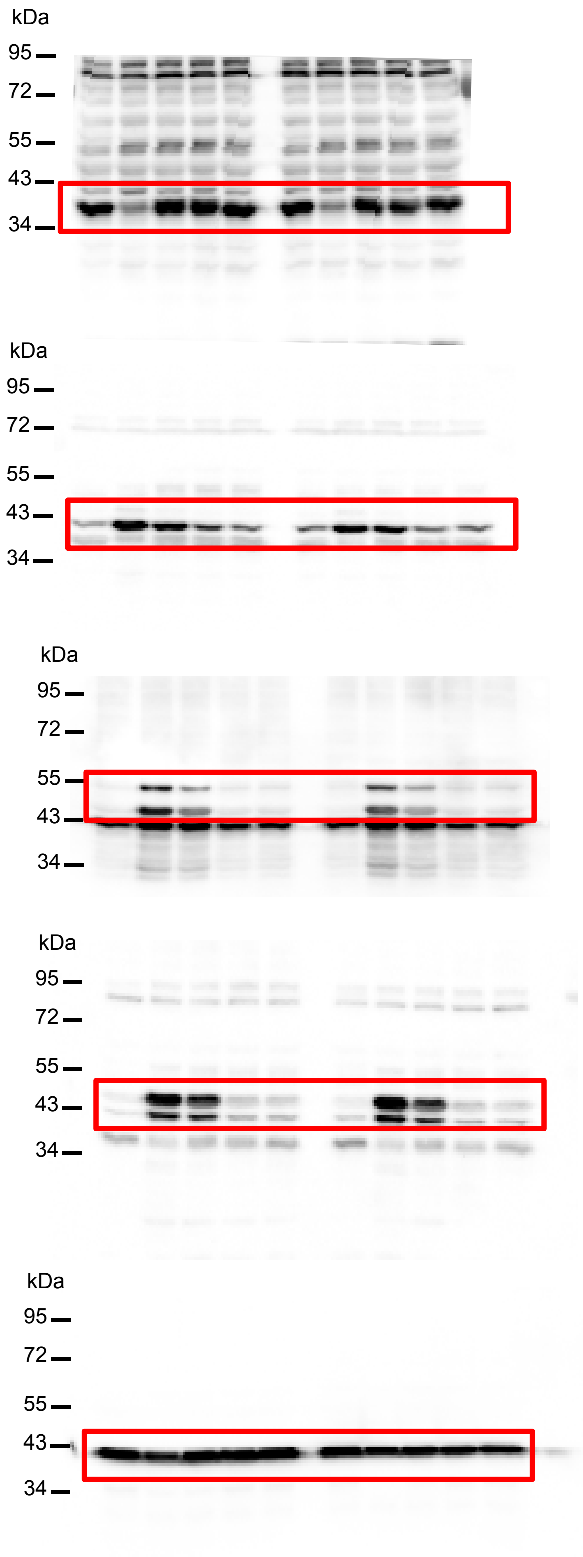

Supplemental Fig. 3A

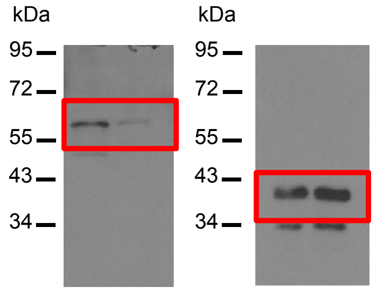

Supplemental Fig. 3D

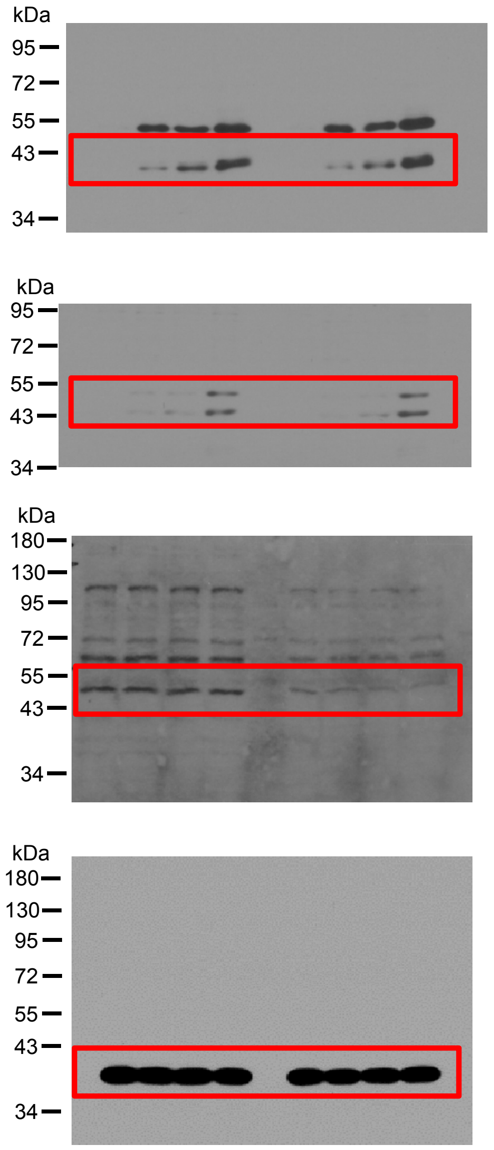

Supplementary Figure 9. Uncropped images of Western blots

Supplemental Fig. 6A

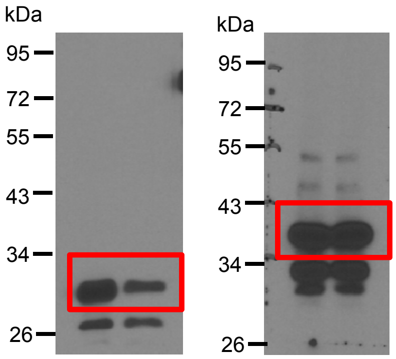

Supplemental Fig. 6C

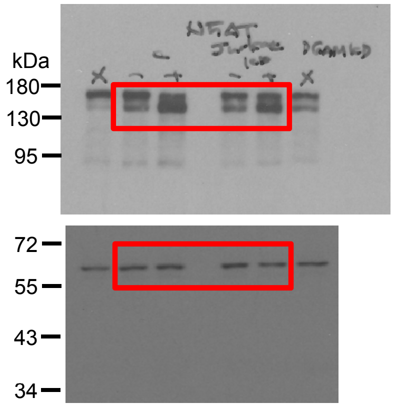

Supplemental Fig. 7B

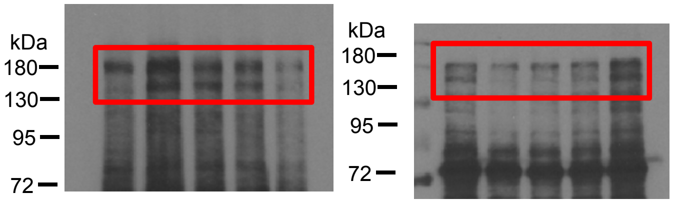

Supplementary Table 1. Quantitative PCR primers

Gene symbol	Sequences (5' → 3')	
	Forward	Reverse
Gapdh	AAGGTCATCCCAGAGCTGAA	CTGCTTCACCACCTTCTTGA
Ifng	GGCCATCAGCAACAACATAAGCGT	TGGGTTGTTGACCTCAAACTTGGC
Tnf	ATGAGAAGTTCCCAAATGGC	CTCCACTTGGTGGTTTGCTA
Il4	AGATGGATGTGCCAAACGTCCTCA	AATATGCGAAGCACCTTGGAAGCC
Tak1	CAGTGTTCCCAAGGAGTGGCTT	CAAGCTCTCAATGGGCTTAGGT
Tab2	AGGACTTTAACACACAGCGT	AAGTTTGATTTCTATCTGGT
Actin	AGTGTGACGTTGACATCCGT	TGCTAGGAGCCAGAGCAGTA

Supplementary Table 2. The sequences of the oligonucleotides used to generate the shRNAs.

Target genes	Sequences (5' → 3')
Mlkl	AAAAGGAGATTCCAAAGGAACATTTGGATCCAAATGTTCCTTTGGAATCTCC
Pgam5-1	AAAAGGAGAAGACGAGTTGACATTTGGATCCAAATGTCAACTCGTCTTCTCCC
Pgam5-2	AAAAGGCTTCATGTTGTCTCAAATTGGATCCAATTTGAGACAACATGAAGCC
Pkcq	AAAAGCAGTTCCTTCATTCCAAATTGGATCCAATTTGGAATGAAGGAACTGC
Pkcg	AAAAGCTAGACTCCTCCAATTATTTGGATCCAAATAATTGGAGGAGTCTAGC
Vav1	AAAAGCACATTCTACCAGGGATATTGGATCCAATATCCCTGGTAGAATGTGC
Tak1	AAAAGCCACAAACGACACTATTATTGGATCCAATAATAGTGTCGTTTGTGGC
Tab2	AAAAGCACATGTGGATAGAATAATTGGATCCAATTATTCTATCCACATGTGC
Drp1	AAAAGCAGAACTCTAGCTGTAATTTGGATCCAAATTACAGCTAGAGTTCTGC
Scramble	AAAAGCTACACTATCGAGCAATTTTGGATCCAAAATTGCTCGATAGTGTAGC
